# “Robust” but Tiny: Methodological Influences and Inter-Individual (Un)Stability of the Serial Order Effect in Creativity

**DOI:** 10.3390/jintelligence14060100

**Published:** 2026-06-04

**Authors:** Baptiste Barbot, Wim Van den Noortgate, Sameh Said-Metwaly

**Affiliations:** 1 Psychological Sciences Research Institute, UCLouvain, 1348 Louvain-la-Neuve, Belgium; 2 Child Study Center, Yale School of Medicine, New Haven, CT 06510, USA; 3 Faculty of Psychology and Educational Sciences, KU Leuven, 8500 Kortrijk, Belgium; wim.vandennoortgate@kuleuven.be (W.V.d.N.); sameh.metwaly@kuleuven.be (S.S.-M.); 4 Imec Research Group, ITEC, 8500 Kortrijk, Belgium; 5 Faculty of Education, Damanhour University, Damanhour 22511, Beheira Governorate, Egypt

**Keywords:** serial order, divergent thinking, creativity, inter-individual stability, reliability, Alternate Use Task, psychological methods, assessment

## Abstract

The serial order effect (SOE), i.e., the tendency for the creative quality of ideas to increase with increasing response position, has been widely documented in divergent thinking (DT) research. However, its magnitude is hardly reported, and its sensitivity to methodological effects (e.g., DT task used, scoring method) has rarely been investigated. More critically, the stability of inter-individual differences in SOE has not been investigated to date. Is the SOE a sizable, dependable (i.e., immune to methodological variations), and importantly, a trait-like psychological phenomenon? This study examined SOE strength and inter-individual consistency across three Alternate Uses Task (AUT) prompts and three scoring approaches: subjective ratings, frequency-based scoring, and semantic similarity. While the SOE emerged across all scoring methods and functional forms examined (linear and quadratic), effect sizes were consistently small in magnitude (explaining at most 3.5% of variance) and varied significantly by stimulus and scoring method. Crucially, evidence for stable individual differences in SOE slopes was absent (ICCs < 0.31 across AUT prompts), raising a potential “reliability paradox”: the SOE appears robust at the group level (i.e., reproducible, though small), yet unreliable in terms of inter-individual stability. These findings underscore the need for systematic evaluation of SOE effect sizes, greater attention to task and scoring differences, and closer investigations clarifying whether the SOE reflects a stable trait interpretable at the individual level, or an aggregate phenomenon with limited practical significance.

## 1. Introduction

The serial order effect (SOE) in Divergent Thinking (DT) is often described as one of the most robust effects observed in creativity studies (e.g., Acar et al., 2019; Bai et al., 2021; Beaty & Silvia, 2012; Gonthier & Besançon, 2024; Hass, 2017; Hass & Beaty, 2018; Kaya & Acar, 2019) or even in modern psychology (Ahn et al., 2023). In a DT task, participants are prompted to generate as many creative ideas as possible in response to a given problem or stimulus within a set time. Stemming from foundational DT work (Christensen et al., 1957), the SOE is observed when the creative quality of measured responses (e.g., originality, response infrequency, semantic distance) increases as response order increases, while the time required to generate these later responses also increases (e.g., Beaty & Silvia, 2012; Hass, 2017; Kaya & Acar, 2019; Wang et al., 2017). In short, it takes more “effort-time” to generate responses of higher creative quality (Barbot, 2018; Taylor & Barbot, 2024). Although the SOE literature is rich, the magnitude of the effect is usually disregarded, and potential moderators of the effect (such as the type of DT task, specific prompt at hand, or creativity scoring method) are rarely investigated systematically. Importantly, there is limited evidence on the stability of the effect at the inter-individual level. In other words, there is little evidence regarding the uniformity of the effect across individuals (e.g., are there subjects more prone to the SOE? Does the SOE reflect a stable problem-solving strategy across different tasks and conditions? In sum, is the SOE a trait-like phenomenon?). After briefly reviewing both pioneering and contemporary research on the SOE, this paper highlights several moderator variables and methodological considerations that may account for variations in the SOE size across studies. It then formally investigates the magnitude and inter-individual stability of the effect across multiple DT prompts and scoring methods, using a published dataset (e.g., Forthmann et al., 2017, 2020; Forthmann & Doebler, 2022).

### 1.1. Classic and Contemporary Interpretation of the SOE

Drawn from Mednick’s (1962) associative theory of the creative process, a classic hypothesis explaining the underlying mechanism of the SOE posits that idea generation progresses in a serial order, moving from the retrieval of common or dominant concepts (early responses) to associations requiring the access of concepts of greater semantic distances (remote/original ideas). That is, the SOE in DT reflects the progressive search for increasingly distant semantic concepts (e.g., Beaty & Silvia, 2012; Wang et al., 2017). Starting from salient or even “fixating” responses (i.e., ideas driven by highly accessible or conventional information due to cognitive constraint arising from previously activated knowledge, examples, or dominant representations, even when these are irrelevant or suboptimal for the task; e.g., Chrysikou et al., 2016; Smith et al., 1993), ideation progressively shifts towards more remote concepts. Consequently, the increased cognitive effort (i.e., effort-time) needed to access ideas of higher creative quality goes hand-in-hand with the slowdown of the response rate as time on task increases (Acar et al., 2018, 2019; Barbot, 2018; Taylor et al., 2021).

However, consistent with dual-pathway models of creativity (e.g., Nijstad et al., 2010), ideation is not merely an issue of the spread of activation to increasingly distant concepts (Beaty & Silvia, 2012); it is also an effortful process over which people have agency (Barbot, 2018; Taylor & Barbot, 2024). Therefore, it is not surprising that much of the contemporary SOE literature has focused on executive functioning in relation to the SOE, including both behavioral (e.g., Bai et al., 2021; Beaty & Silvia, 2012; Cheng et al., 2016) and neuroscience studies examining the involvement of “executive” cortical regions in the SOE (e.g., Heinonen et al., 2016; Kraus et al., 2019; Wang et al., 2017). In a nutshell, specific executive functions are required to sustain the creative search. If early ideas are often retrieved directly from memory, later and more original ideas are based on strategies involving greater cognitive effort (Gilhooly et al., 2007). Such top-down control processes include shifting (Hubert et al., 2025; Wang et al., 2017) and inhibition (Cassotti et al., 2016; Cheng et al., 2016) whereby obvious or common ideas are suppressed, while strategically switching to new idea categories as the task progresses (hence allowing more original ideas to appear later). 

In sum, current understanding of the SOE acknowledges both associative mechanisms recruiting “salient” associations at the beginning of an ideation sequence and, subsequently, more effortful, top-down control processes supporting the search for more distant ideas.

### 1.2. Moderators of the SOE

In line with the associative and executive interpretations of the SOE, several studies have investigated the role of various cognitive factors as predictors or moderators of the SOE in creativity. To name a few, the role of fluid intelligence (G*f*) has been outlined as a moderator of the SOE (Beaty & Silvia, 2012; Hass, 2017). Findings have shown that people with high G*f* are better equipped to engage strategic retrieval and cognitive control processes earlier in DT ideation (i.e., recruiting executive functioning), whereas low G*f* individuals may initially rely more on the passive spreading of activation and struggle to strategically guide their search, resulting in a steeper SOE slope (although contradictory findings exist; see Miroshnik & Shcherbakova, 2019; Runco, 1986). Similarly, Hubert et al. (2025) showed how individual differences in executive shifting abilities shape the SOE (i.e., the functional form of the SOE). Because executive functioning develops sharply from childhood to early adulthood (e.g., Diamond, 2013; Maldonado et al., 2020; Zelazo & Carlson, 2012), and differential patterns of gender-related differences in executive functioning have been shown (e.g., Grissom & Reyes, 2019), it is expected that gender and age could moderate the SOE, reflecting distinct cognitive strategies in DT. However, these factors are rarely accounted for in SOE research, while both age-related and gender-related differences in DT performance have been confirmed meta-analytically (Said-Metwaly et al., 2021; Taylor et al., 2024). In a series of SOE studies by Bai et al. (2021, 2024), the authors replicate a SOE with the same sample of children at 4 and 6 years old, but did not examine explicitly age-related differences in SOE nor the stability of inter-individual differences in SOE over time.

Recently, Gonthier and Besançon (2024) highlighted that the SOE is tightly linked to the ideation sequence length (i.e., ideational fluency effect): while lower fluency was associated with higher originality of responses regardless of serial position, higher fluency was associated with a stronger SOE, though never reaching the level of originality of lower ideational fluency individuals. The authors interpreted this finding in light of the quantity-quality trade-off in DT (e.g., Forthmann et al., 2020), whereby DT response strategies vary, either emphasizing fluency (many responses of lower originality) or originality (fewer responses of higher originality). Importantly, their study also highlighted that findings on the SOE varied in magnitude according to the task considered. This moderating effect of the task may lie in differences in task-specific “solution space”, defined as the total set of mental representations and associations that an individual considers accessible or relevant for a given task (e.g., Forthmann et al., 2020). From this perspective, tasks with a narrower solution space may constrain ideational sequences by limiting the range of representations that can be explored during idea generation. However, akin to most of the SOE literature, this study focused on establishing the phenomenon’s existence and examining cognitive moderators at the aggregate (group) level, leaving critical methodological questions regarding the reliability and magnitude of the effect across experimental conditions largely unaddressed.

### 1.3. Methodological Influences on the SOE 

The creativity research literature offers a rich body of evidence on the effect of instruction, task/prompt, and scoring methods on DT performance (e.g., Forthmann et al., 2016; Harrington David, 1975; Reiter-Palmon et al., 2019; Runco et al., 2005; Said-Metwaly et al., 2020; Silvia et al., 2008), often appearing as significant moderators in meta-analyses (e.g., Said-Metwaly et al., 2021, 2022; Taylor et al., 2024). In studies that have employed various tasks or stimuli, differences in the SOE magnitude across conditions often transpire (Christensen et al., 1957; Gonthier & Besançon, 2024; Runco, 1986), although these methodological effects are rarely formally compared or discussed. 

Perhaps one of the most striking findings in this line of research is a “reverse” SOE (Ahn et al., 2023) observed in hybrid brainstorming settings, where individuals generate ideas alone first, then share them in a group. In this context, the quality of ideas shared peaks early and then declines. This reversal was theorized to occur due to impression management: individuals prioritize sharing their *best* ideas first to establish competence or influence the group, overriding the “typical” cognitive sequence (Ahn et al., 2023). Similarly, external cues, such as examples (de Chantal et al., 2025) or explicit instructions to be original can modulate the typical sequence of cognitive strategy use in DT, making individuals more likely to engage executive functions (Kaya & Acar, 2019). Specifically, these instructions can push the production of highly elaborate, more original ideas into earlier phases, although the fundamental sequence (i.e., SOE) is not eliminated (Kaya & Acar, 2019). However, a study comparing the effect of “be-fluent” versus “be-creative” instructions in DT revealed only a weak moderating effect of DT instruction on the SOE (Kleinkorres et al., 2021).

Regarding DT scoring methods effects, although the SOE has already been demonstrated using semantic distance scoring in studies also including human judgments and other scoring methods, formal comparisons of the SOE across these different methods are generally lacking (e.g., Beaty & Johnson, 2021; Hass, 2017). An exception is a line of work specifically investigating self-assessment of creative ideas in DT tasks (e.g., Kenett et al., 2023; Miroshnik & Shcherbakova, 2019; Sidi et al., 2020), although this method probably captures metacognitive processes and self-efficacy more accurately than it reflects the creative quality of DT responses. This research line shows that self-assessment of creative ideas generally aligns with the SOE (Kenett et al., 2023; Miroshnik & Shcherbakova, 2019; Sidi et al., 2020), and more so than actual originality scores (whether based on human judgments or semantic distance; Kenett et al., 2023; Sidi et al., 2020).

Finally, several SOE studies have employed multiple task types or stimuli, but have generally aggregated conditions when reporting overall findings (e.g., Kleinkorres et al., 2021; Wang et al., 2017). However, much research has shown that DT performance is sensitive to the task, or even the stimulus at hand (for review, see Barbot, 2018; Barbot et al., 2020). Indeed, creativity does not happen “in a vacuum”, and individual differences in experience with the task at hand may impact performance (Runco et al., 2006). In this line, Kaya and Acar (2019) report that early ideas tend to come from experiences and late ideas are more likely to be inexperienced (consistent with the executive interpretation), with effect sizes varying according to the prompt and instructions used (with estimated explained variance ranging from 0.03% to 14.7%).

### 1.4. How Sizable Is the SOE?

As briefly illustrated in the succinct review above, the SOE in DT has sparked enthusiasm in the creativity research literature and contributed to a better understanding of creative thinking processes and their underlying mechanisms. Prolific research efforts continue to fuel this literature (de Chantal et al., 2025; Gonthier & Besançon, 2024; Hubert et al., 2025; Raz et al., 2025). However, a noticeable finding in SOE research (yet rarely presented or discussed explicitly) is the rather small or even trivial effect sizes associated with the SOE (when reported at all). Indeed, much of the SOE literature seems to disregard the magnitude of the SOE observed, and the reported statistics are often insufficient to estimate effect sizes (e.g., Beaty & Johnson, 2021; Heinonen et al., 2016; Hubert et al., 2025; Kleinkorres et al., 2021). When a “significant” SOE is reported—perhaps unsurprisingly, given that null findings are less likely to be discussed (although see Raz et al., 2025 for an exception)—the associated effect sizes are often strikingly underwhelming. 

For example, Miroshnik and Shcherbakova (2019) reported a *R*^2^ of 0.009 for (linear) SOE based on self-assessed judgment of old/new ideas, and *R*^2^ = 0.008 based on expert judgment, whereas quadratic SOE models improved model fit, with *R*^2^ = 0.038 and 0.022 for self-assessment and expert judgment, respectively—hence, at most, 3.8% of explained variance across conditions and SOE curves. Likewise, an EEG study by Kraus et al. (2019) reported a SOE across three DT stimuli of similarly small magnitude (1.6% variance explained). Though not always straightforward to derive from reported statistics, many other examples from the SOE literature suggest effect sizes of very small magnitude across various experimental contexts. Starting with the classic Beaty and Silvia (2012) study that has inspired contemporary SOE research, the reported (linear) SOE in creativity converts into an approximately 5% explained variance, while other studies reporting significant SOE convert into negligible effect sizes (e.g., Gonthier & Besançon, 2024, <1%; Hass, 2017, <2%). A notable exception (de Chantal et al., 2025) suggests an overall SOE of semantic distance, translating into about 14% explained variance, in a study exposing participants to different types of examples across three DT prompts.

Together, while modest effect sizes can be meaningful in certain psychological contexts (for instance, when the outcome has high real-world impact or when the effect accumulates over time) they also invite closer examination of moderators that may account for variability in the magnitude of the SOE. That is, even an overall small effect may become considerable in specific conditions or subpopulations, making moderator analysis a valuable next step. In particular, the generally low SOE magnitude reported in the literature raises the question of whether the aggregate effect masks substantial inter-individual differences—that is, whether individual SOE slopes reflect a reliable, trait-like characteristic. When an effect exhibits limited explanatory power at the population level, this may signal unmodeled heterogeneity, thereby inviting scrutiny of the stability and reproducibility of individual differences. Yet, despite a rich body of empirical studies employing multiple DT tasks, instructions, and scoring methods, questions concerning variation in effect magnitude and the stability of the SOE across individuals and conditions have received surprisingly limited attention, with no studies to date formally examining these issues across such methodological contexts.

### 1.5. Present Study 

Despite the apparent robustness of the SOE reflected in the reproducibility of group-level effects across studies examining the temporal dynamics of DT responses, relatively few studies have explicitly addressed the typically modest magnitude of the effect, examined variations in effect size as a function of scoring methods or task characteristics, or investigated whether inter-individual differences in the SOE are stable across tasks and scoring approaches (i.e., whether the SOE reflects a uniform, trait-like tendency). Accordingly, the present study addresses these gaps through an empirical investigation comparing the magnitude and stability of the SOE across three DT stimuli and three distinct scoring methods (frequency-based scoring, subjective human ratings, and semantic similarity). Specifically, this study pursues two aims: (1) to evaluate the magnitude of the SOE across different stimuli and scoring approaches, and (2) to assess the stability of individual differences in the SOE across DT stimuli and scoring methods. By doing so, we aim to contribute to a broader discussion of methodological moderators that may shape SOE findings in the creativity literature, and to critically examine both the magnitude and the extent to which the SOE represents a robust group-level phenomenon and/or a stable inter-individual characteristic.

## 2. Methods

### 2.1. Participants 

The data used in the present study (available here: https://osf.io/a9qnc; accessed on 2 February 2025) stem from a dataset collected in Germany and analyzed in several prior publications by the original team (e.g., Forthmann et al., 2017; Forthmann & Doebler, 2022) and recent secondary analyses conducted as part of the current Special Issue^1^ (e.g., Jendryczko, 2024; Myszkowski & Storme, 2025; Pellegrino et al., 2025; Weiss et al., 2025). The original dataset comprised 202 participants, including 142 women and 58 men, 78% of whom were university students. Their age ranged from 17 to 75 years, with a mean age of 24.5 (SD = 6.8). Further information on the sample can be found in the previous publications utilizing this dataset (Forthmann et al., 2017, 2020; Forthmann & Doebler, 2022).

### 2.2. Material and Procedures

DT was assessed using three Alternate Uses Tasks (AUT; e.g., Guilford, 1967). In AUTs, participants are typically asked to generate as many creative uses as possible for a common object within a set time. Although this task is not a universal operationalization of DT, it is by far the most widely used in creativity research (Saretzki et al., 2024) and was recently found to be the most central operationalization among 27 different DT tasks (Barbot et al., 2026). In the data analyzed for the present study, three objects (i.e., prompts) commonly used in the literature (Wallach & Kogan, 1965) were employed: *garbage bag*, *paperclip*, and *rope*. For each prompt, participants had 2.5 min to generate alternate uses using “hybrid instructions”, which emphasize both fluency (quantity of ideas) and originality (quality of ideas) as core dimensions of divergent thinking (Reiter-Palmon et al., 2019). The instructions read: “*Please try to write down as many uncommon and creative uses for a [object-prompt] as you can think of*” (Forthmann et al., 2017). While such hybrid instructions have been shown to increase creative performance (e.g., Butler et al., 2003), they might also emphasize individual “preferences” for either fluency (i.e., quantity) or originality (i.e., quality; e.g., Torrance, 1998).

In total, 4752 individual responses were recorded (1648 for *garbage bag*, 1429 for *paperclip*, and 1675 for *rope*). Responses were scored for fluency (the count of valid responses generated per prompt), flexibility (the number of distinct response categories generated per prompt), and originality. To score the latter, three raters evaluated each individual response for creative quality (thereafter subjective judgment) representing its overall uncommonness, remoteness, and cleverness (see Silvia et al., 2008). Ratings were then aggregated at the response level with high inter-rater reliability (ICC(3,k) = 0.86, 0.84, and 0.91 for *Garbage bag*, *paperclip,* and *rope*, respectively). Additionally, the classic frequency-based scoring was applied (e.g., Wilson et al., 1953), providing the relative frequency of each response in the participant sample (i.e., weighting each response by its frequency of occurrence in the total pool of responses gathered in the sample). Finally, semantic similarity scores obtained from the unweighted dewak GloVe full semantic space (Forthmann & Doebler, 2022) were calculated at the response level. Note that, akin to the frequency-based scoring method, higher values reflect responses of lower creativity (i.e., here more semantically similar). For more information on scoring methods and procedures, see Forthmann et al. (2017, 2020) and Forthmann and Doebler (2022).

### 2.3. Analyses

All statistical analyses were performed in *R* (version 4.2.2). Because response creativity was evaluated using three different scoring methods (i.e., subjective judgment, frequency-based scoring, and semantic similarity), all analyses were conducted separately for each method. To assess how response-level creativity changed as a function of response order, we fitted two-level linear mixed-effects models with random intercepts and random slopes, estimated via restricted maximum likelihood using the *nlme* package (Pinheiro et al., 2025). The models included response order as the focal predictor and controlled for stimulus type (i.e., AUT object-prompt), participants’ fluency (in line with Gonthier & Besançon, 2024), as well as gender and age, which have been shown to relate to variability in creative performance (Said-Metwaly et al., 2021; Taylor et al., 2024), while accounting for the hierarchical structure of responses nested within participants. To further examine inter-individual differences in SOE (according to the prompt and scoring method), we extracted participant-level slopes for the effect of response order on response creativity using empirical Bayes estimates. These estimates combine information from both the individual and the full sample, thereby providing more robust estimates (Raudenbush & Bryk, 2002).

To investigate whether the effect of response order varied depending on AUT object-prompt, fluency score, flexibility score, gender, or age, each considered moderator was tested by including a separate two-way interaction term (response order × moderator) in the models. The significance of these interaction effects was assessed using a Type III analysis of variance, with degrees of freedom estimated via Satterthwaite’s method (Satterthwaite, 1946). For categorical variables (i.e., AUT object-prompt and gender), significant interactions were followed up with post hoc comparisons using Bonferroni adjustments via the *emmeans* package (Lenth, 2023). The size of the observed effects was quantified using Cohen’s *d*, calculated with the *effectsize* package (Ben-Shachar et al., 2020) and interpreted according to conventional guidelines (Cohen, 1988). Effects were visualized with the *ggeffects* package (Lüdecke, 2018). 

Consistent with previous work suggesting a better fit of SOE patterns with quadratic slopes (Beaty & Silvia, 2012; Hass & Beaty, 2018; Miroshnik & Shcherbakova, 2019), two-level quadratic regression models were fitted to assess whether a nonlinear relationship better described the effect of response order on response creativity. These models incorporated all predictors from the linear models and added a squared term for response order. Model fit was compared to the linear model using a likelihood ratio test to determine whether the quadratic term significantly improved fit. Potential moderator variables were examined by including interaction terms with both the linear and quadratic components of response order.

Finally, to gauge the extent to which the SOE is reproducible across scoring conditions and prompts in terms of inter-individual stability (rather than group-level, average effect), intraclass correlation coefficients (ICC[3,k]; Koo & Li, 2016) were computed, using the *psych* package (Revelle, 2025), on slope parameters extracted from models predicting response creativity from response order, fitted separately for each combination of participant and prompt, and this, for each scoring method. Interpretation of ICC values followed the guidelines proposed by Koo and Li (2016). 

## 3. Results

### 3.1. Results of Linear Regression Analysis

#### 3.1.1. Overall Analyses

**Figure 1:**
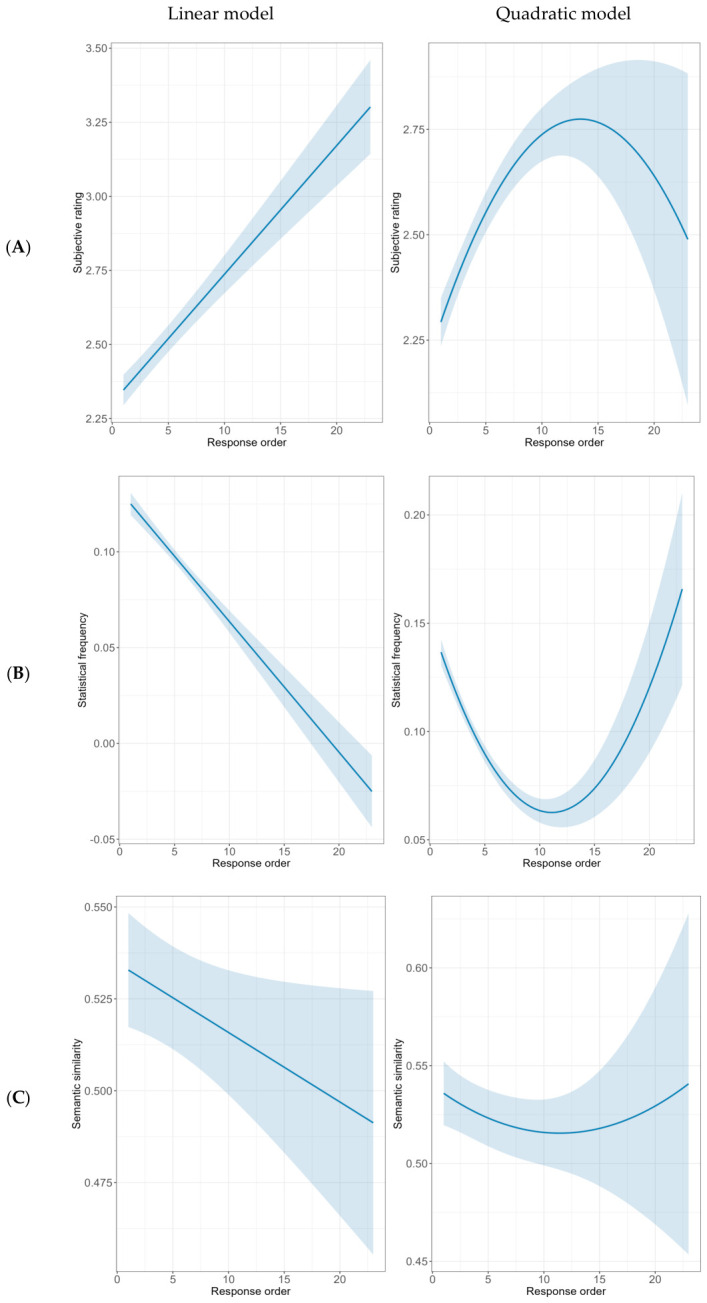
Predicted Response Creativity by Response Order for Linear and Quadratic Regression Models across the Three Scoring Methods (Panel (**A**): Subjective rating, Panel (**B**): Statistical frequency; Panel (**C**): Semantic similarity).

Electronic Supplementary Materials (ESM) Table S1 summarizes the parameter estimates, their standard errors, and associated *p*-values for all linear regression models across the three scoring methods. Model 1 (see Table 1) showed that response creativity (or its inverse according to whether subjective judgment, response frequency, or semantic similarity is considered) significantly increased with response order, although this pattern manifested differently depending on the scoring metric used (see Figure 1, left panels). When using subjective judgment, later responses were rated as more creative (*b* = 0.15, SE = 0.01, *p* < .001), such that a one standard deviation increase in response order (SD = 3.54) corresponded to an increase of approximately 0.15 units in creativity (i.e., for every one raw unit increase in response order, creativity scores rise by about 0.04 points, or about 1.58% of the sample mean). For frequency-based scoring, later responses were less frequent (*b* = −0.02, SE = 0.002, *p* < .001), such that each one raw unit increase in response position corresponded to a decrease of about 0.01 units in frequency scores, or roughly 10% of the sample mean. For semantic similarity, later responses were more semantically distant (*b* = −0.01, SE = 0.003, *p* = .04), such that each one raw unit increase in response order corresponded to an increase of about 0.003 units in semantic distance, or approximately 0.58% of the sample mean.

Although the SOEs were statistically significant, their magnitudes were small (Cohen’s *d* = 0.31, 0.38, and 0.06 for subjective judgment, frequency-based scoring, and semantic similarity, respectively), and the proportion of variance explained was modest (partial *η*^2^ translating into 2.35%, 3.53%, and 0.10%, respectively), suggesting that response order has a rather minor impact on response creativity. The empirical Bayes estimates of the SOE formed unimodal distributions (see Figure S1 in ESM) with clear central clustering and noticeable dispersion, reflecting variability among participants beyond the central tendency.

#### 3.1.2. Moderator Analyses

For subjective judgment scoring, among the tested moderator variables (Models 2 to 6; Table S1), only fluency and flexibility showed significant effects. Both moderator variables exhibited negative interactions with response order, indicating that the positive effect of later responses on creativity diminishes as fluency or flexibility increases. In other words, participants with higher fluency or flexibility show smaller gains in creativity for later responses compared to those with lower fluency or flexibility.

**Figure 2:**
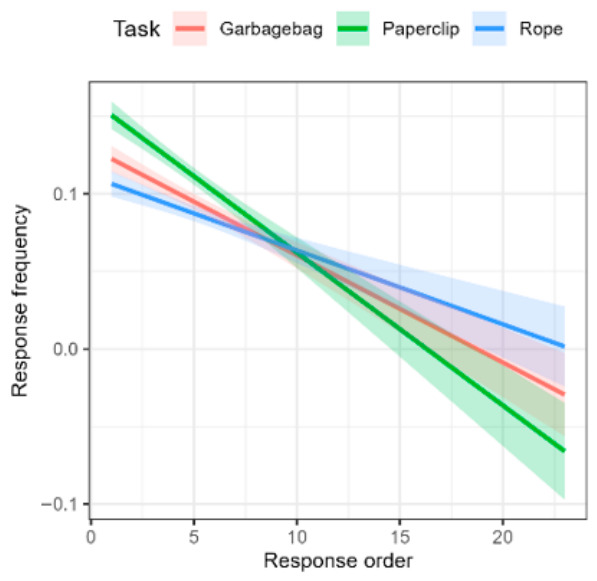
Predicted Response Frequency by Response Order and AUT-Prompt for the Linear Regression Model.

For frequency-based scoring, only stimulus type, fluency, and flexibility showed significant moderating effects. As illustrated in Figure 2, the negative impact of response order on response frequency varied by stimulus, being strongest for the *Paperclip* prompt (*b* = –0.035), followed by the *Garbage bag* prompt (*b* = –0.024), and weakest for the *Rope* prompt (*b* = –0.017). Except for the comparison between the *Garbage bag* and *Rope* prompt (Bonferroni *p* = .07), the other two comparisons were statistically significant (Bonferroni *p* = .01 for the comparison between *Garbage bag* and *Paperclip*, and *p* < .001 for the comparison between *Paperclip* and *Rope*). Both fluency and flexibility moderated the response order effect positively (See Table S1 and Figure S2 in ESM): higher fluency or flexibility attenuated the SOE, so that participants with higher fluency or flexibility generated responses with consistent response frequency across the sequence, whereas those with lower fluency or flexibility showed sharper decreases in response frequency for later responses.

For semantic similarity scoring, none of the tested moderator variables showed a significant effect. 

### 3.2. Results of Quadratic Regression Analysis

#### 3.2.1. Overall Analysis

The full quadratic model (i.e., including all predictors and covariates) provided a significantly better fit than the full linear model for all three scoring methods (subjective judgment: χ^2^ = 11.75, *p* < .001; frequency-based scoring: χ^2^ = 54.23, *p* < .001; semantic similarity: χ^2^ = 9.20, *p* = .002; See Table S2 in ESM). Despite this improvement in fit, both models explained almost the same amount of variance, with nearly identical *R*^2^ values translating into 4.99% for subjective judgment, 7.41% for frequency-based scoring, and 4.28% for semantic similarity. However, the proportion of variance explained by the quadratic effect only (i.e., quadratic SOE) was modest across the three scoring methods and even smaller than that for the linear effect (partial *η*^2^ = 0.43% for subjective judgment, 1.67% for frequency-based scoring, and 0.03% for semantic similarity, respectively). Because the added complexity of the quadratic model does not translate into meaningful improvements in explained variance, the linear model results remain the most interpretable and practically relevant, and the quadratic results are presented only for completeness.

As shown in Table S2 (ESM) and the right panels of Figure 1, the quadratic model (Model 7) indicated a significant quadratic effect of response order for the subjective judgment and frequency-based scoring methods, whereas the quadratic effect was not significant for the semantic similarity scoring method. For subjective judgment scoring, the quadratic coefficient was negative: creativity increases for later responses, but this increase slows and eventually begins to reverse towards the last responses, yielding a concave downward curve. Coherently, for frequency-based scoring, the quadratic coefficient was positive: response frequency decreases across the sequence, but this decline slows and begins to reverse for the final responses, producing a concave upward pattern.

#### 3.2.2. Moderator Analyses

For subjective judgment scoring, among the tested moderator variables (Models 8–12 in ESM Table S2 and Figure S3), only fluency and flexibility significantly moderated the quadratic effect of response order. The negative quadratic effect of response order was weaker for participants with higher fluency and flexibility, indicating that participants with greater fluency or flexibility experienced a smaller decline in creativity toward the end of the response sequence.

For frequency-based scoring, fluency, flexibility, and age showed significant moderating effects. Higher fluency and flexibility were associated with a weaker quadratic effect, with response frequency remaining more consistent across the sequence, whereas lower fluency or flexibility was associated with a steeper drop early on and levelling off towards the end of the sequence. Younger participants exhibited a stronger quadratic effect, which diminished with age.

For semantic similarity scoring, none of the tested moderator variables showed a significant effect. 

### 3.3. Inter-Individual Stability of SOE

**Figure 3:**
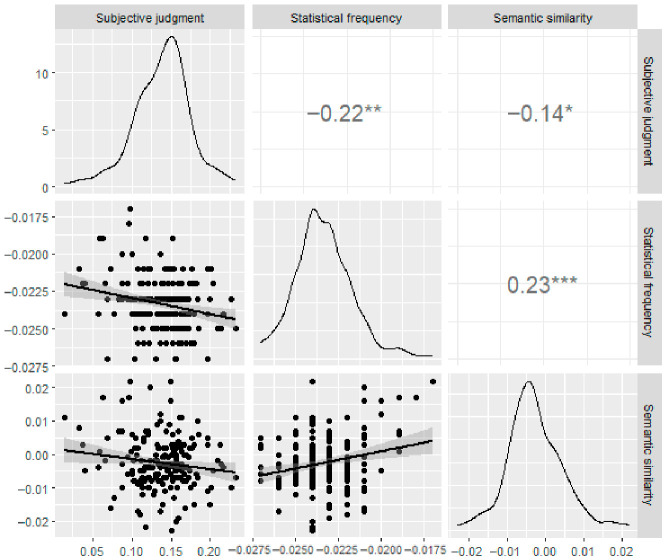
Correlations among Empirical Bayes Estimates of the SOE across the three scoring methods. *: *p* < .05, **: *p* < .01, ***: *p* < .001.

To examine the consistency of the SOE across the three scoring methods, we analyzed the correlations among empirical Bayes estimates for the effect of response order (i.e., across AUT object prompts). The correlations were uniformly small, ranging from |0.14| to |0.23| (Figure 3), and although statistically significant, their magnitudes indicate that the scoring methods capture largely distinct sequential patterns. However, despite differences in magnitude and sign, all three scoring methods showed a consistent directional pattern, with later responses being more creative, as indicated by higher subjective creativity ratings and lower frequency and semantic similarity.

Furthermore, intraclass correlation coefficients (ICC(k,3)) of empirical Bayes estimates obtained across scoring methods and stimuli suggested unreliable inter-individual differences in the SOE according to the scoring method for *garbage bag* (ICC_[95% CI]_ = 0.00 [−0.26, 0.22]), *paperclip* (ICC_[95% CI]_ = 0.28 [0.09, 0.44]), and *rope* (ICC_[95% CI]_ = 0.19 [−0.02, 0.37])^2^.

Similarly, unreliable inter-individual differences in the SOE according to the prompt used were found for subjective judgment (ICC_[95% CI]_ = 0.31 [0.12, 0.46]), response frequency (ICC_[95% CI]_ = 0.25 [0.05, 0.41]), and semantic similarity (ICC_[95% CI]_ = 0.00 [−0.27, 0.22]). For contrast, intraclass correlation coefficients (ICC(k,3)) were computed using the classic DT indexes across prompts. Both fluency and flexibility yielded excellent inter-individual stability across prompts (both ICC_[95% CI]_ = 0.84 [0.80, 0.88]). Originality indexes showed good stability for subjective ratings (ICC_[95% CI]_ = 0.67 [0.58, 0.74]) and semantic similarity (ICC_[95% CI]_ = 0.80 [−0.75, 0.84]), whereas frequency-based scoring showed poor stability (ICC_[95% CI]_ = 0.45 [0.30, 0.57]).

## 4. Discussion

### 4.1. Is the SOE a Robust Group-Level Finding?

Confirming the robustness of the SOE described in the DT literature (e.g., Acar et al., 2019; Ahn et al., 2023; Bai et al., 2021; Beaty & Silvia, 2012; Gonthier & Besançon, 2024; Hass, 2017; Hass & Beaty, 2018; Kaya & Acar, 2019), this study demonstrated the (group-level) reproducibility of the SOE in a dataset involving three DT stimuli and three scoring methods. However, unlike most work in this line, the present study focused on gauging the magnitude of the effect and scrutinized methodological moderators explaining variations in the effect sizes observed. As anticipated from our brief survey of recent SOE research, our findings confirmed that this magnitude is overall negligible to small. Specifically, while response order explained only up to 3.53% of the variance in response creativity in linear models, the quadratic term explained only an additional 1.67% of variance beyond the linear effect, despite improved model fit.

First, this finding is consistent with prior research identifying a superior fit of nonlinear SOE models over linear models (e.g., Beaty & Silvia, 2012; Gonthier & Besançon, 2024; Kleinkorres et al., 2021; Miroshnik & Shcherbakova, 2019), such that the “typical” (i.e., average) creativity sequence in DT tends to plateau (or even slightly decrease) towards the end of the sequence. Despite the superior fit of non-linear models in our study, the similarity in explained variance between linear and quadratic models suggests that linear models may remain a parsimonious and practically relevant approach to studying the SOE.

Second, the variations of the SOE’s effect size according to the scoring method examined in this study provide new insights, as they have rarely been formally examined. Regardless of the functional form of SOE or DT stimulus considered, our findings suggest that the SOE is stronger when scoring DT responses with frequency-based scoring (3.53% and 0.43%, for linear and quadratic SOE) than with subjective human judgment (2.35% and 1.67%), or semantic similarity (0.10% and 0.03%). This finding is somewhat surprising, as both theoretical (Mednick, 1962) and empirical grounds (Beaty & Johnson, 2021; Hass, 2017) explain the SOE in DT tasks partly as an increasing spreading of activation to more distant semantic concepts, which should translate into an increasing semantic similarity as response order increases. The same is not necessarily expected for subjective human judgment, because a highly remote or infrequent response does not automatically make it more creative (e.g., Golab & Barbot, 2024). Coherently, the intercorrelations between empirical Bayes estimates (of SOE slopes) across scoring methods were uniformly small (i.e., at most 5.3% shared variance), suggesting that the SOE captures largely distinct sequential patterns across scoring methods. With that said, the stronger effect observed with the frequency-based scoring method may be partly artifactual. Specifically, the high prevalence of common, fixating responses in early ideation may disproportionately amplify the SOE when scored via frequency-based scoring, a methodological bias further discussed in this section. Adding to this unexpected finding in our study, one of the largest SOE effect sizes (14%) reported in our introductory review (de Chantal et al., 2025) was observed using semantic distance. In contrast to that study, which used a “*be creative*” instruction, the data analyzed in the present study were based on hybrid DT instructions (e.g., Reiter-Palmon et al., 2019), which emphasize both fluency and originality. Although research suggests an attenuated SOE when “*be creative*/*be original*” instructions are prompted over “standard” or “*be fluent*” instructions (Kaya & Acar, 2019; Kleinkorres et al., 2021), hybrid instructions might emphasize individual “preferences” for fluency versus originality (i.e., the quantity-quality trade-off), which has been shown to overshadow the SOE (Gonthier & Besançon, 2024). 

Perhaps another way to enlighten this finding is to consider the stronger SOE effect observed with the frequency-based scoring across all three DT prompts. Because relatively few SOE studies have used this otherwise classic scoring approach (Wilson et al., 1953), it is difficult to situate this finding within the existing literature. One plausible interpretation, however, follows from a known limitation of frequency-based scoring noted by Silvia et al. (2008; see also Hocevar, 1979): the base rate of “unique” ideas is sample-size dependent, such that larger samples raise the threshold for receiving high weights. Reciprocally, salient familiar ideas and potentially fixating responses, which are more likely to occur early in the idea generation sequence (see, e.g., “path to least resistance”; Ward, 1994) will receive disproportionately higher frequency weights relative to later, less frequent ideas. This contrast may exert disproportionate leverage on the SOE slope, artificially steepening the decline over serial position. In other words, this scoring approach could accentuate the SOE, conflating between-person base-rate effects (overweighting common ideas) and individual-level trajectories. This mechanism might be less at play when using scoring methods that are less sensitive to base rates, such as subjective scoring (Silvia et al., 2008) or semantic distance (Hass, 2017). 

Along the same lines, our study detected a significant moderation effect of the DT stimulus (i.e., AUT object prompt) when using frequency-based scoring, with the strongest SOE observed for the *paperclip* prompt and the weakest effect for the *rope* prompt. From the base-rate interpretation outlined above, this pattern is noteworthy because the paperclip prompt elicited the fewest total responses in the dataset (*i* = 1429), whereas the rope prompt elicited the most (*i* = 1675), suggesting differences in their “solution space” size (note that the solution space of these three items has been thoroughly analyzed using this sample in related work; see Forthmann et al., 2020). Such differences in solution space may be related to object frequency (Forthmann et al., 2016), prior experience with the stimuli (e.g., Dahlman et al., 2013; Runco et al., 2006), and critically, the prominence of dominant or fixating responses (Gilhooly et al., 2007). Though the solution space (total number of answers) is only an indirect proxy for base-rate concentration, stimuli associated with concentrated base-rate distributions (i.e., presence of several fixating responses) are likely to yield greater weight to early, common ideas, thereby increasing their leverage on SOE slope estimates. Smaller or more constrained solution spaces (e.g., *paperclip*) could yield more dominant, fixating responses, such that a broader range of readily available responses easily retrievable from memory (Kaya & Acar, 2019; Ward, 1994) may be generated early on, delaying more effortful processes that could produce the more infrequent responses. These early, high base-rate responses may, in turn, increase the weight of early responses when estimating SOE slopes. In sum, the DT stimulus moderation effect of the SOE may reflect, at least in part, a scoring × stimulus interaction driven by the presence of dominant/fixating answers. Consistent with this interpretation, an examination of density plots for the three object-prompts (ESM Figure S4) suggests that the *paperclip* prompt is associated with several dominant/fixating responses (i.e., frequencies > 30%), whereas the *rope* prompt is more evenly distributed with no salient, predominant fixating response. Further, the moderating effect of DT stimulus was not observed with the other scoring methods that are less sensitive to base-rate.

Regarding the moderators examined in this study, both fluency and flexibility positively moderated the SOE when originality was scored using frequency-based and subjective judgment methods, but not semantic similarity. Specifically, lower fluency or flexibility was associated with a steeper SOE. Although this finding is also consistent with the base-rate interpretation outlined above, this pattern highlights the influence of ideation sequence length (e.g., Gonthier & Besançon, 2024) and the potential role of cognitive shifting (Hubert et al., 2025; Wang et al., 2017)—mapping with flexibility in DT—in shaping the magnitude of the SOE. Finally, age moderated the SOE only when using frequency-based scoring and non-linear models. This first suggests that the effect of age was not consistent in explaining differences in the SOE, which may be due to the rather homogeneous nature of the sample (i.e., mostly young adults). Although limited literature can support interpretation for this finding (Bai et al., 2021, 2024), it may reflect developmental trajectories in creative cognition. Specifically, younger individuals may be at a stage where idea generation is more strongly shaped by early-emerging, highly accessible responses, leading to a stronger SOE, whereas with increasing age, creative responses may become more evenly distributed across the ideation sequence as cognitive control, strategy use, and experience continue to develop (Barbot & Tinio, 2015).

### 4.2. Inter-Individual Stability of the SOE: A “Reliability Paradox”? 

An important contribution of the present study was to examine for the first time the inter-individual stability of the SOE across DT prompts and scoring approaches. Although a “robust” SOE consistently emerged at the group level, inter-individual differences in SOE slopes were largely unstable across prompts. As a concrete illustration, a participant showing a strong SOE for the *paperclip* prompt was not more likely to show a strong SOE for the *rope* prompt. This pattern suggests that an individual’s SOE slope does not reflect a uniform, trait-like tendency, at least in the present dataset and under the slope estimation methods used (empirical Bayes shrinkage provides regularized, less noisy estimates, so any observed stability should be interpreted as conservative; Raudenbush & Bryk, 2002). This finding stands in marked contrast to the high reliability observed for classic DT indices (i.e., fluency and flexibility) estimated across prompts, as well as the generally good reliability of originality scores themselves (except for the frequency-based scoring approach, which may be affected by the base-rate issue discussed herein; see Forthmann et al., 2020).

Slope parameters have been shown to be reliable individual-difference measures in domains such as cognition, learning, and perception (e.g., Ackerman, 1987; Barbot et al., 2016; Wichmann & Hill, 2001). Why, then, do SOE slopes in DT appear psychometrically fragile? One likely explanation concerns the intensity of measurement occasions. Reliable slope estimates typically rely on paradigms with dense repeated observations, whereas SOE slopes in DT are derived from relatively short ideation sequences (especially in the case of lower fluency). Furthermore, SOE slopes may be especially sensitive to measurement noise introduced by scoring methods (especially frequency-based scoring, as illustrated in the present study) and to large response-to-response fluctuations. Such fluctuations may reflect the concurrent engagement of multiple cognitive strategies such as memory retrieval (e.g., Ward, 1994), shifting (Hubert et al., 2025; Wang et al., 2017), or selective attention (e.g., Bai et al., 2021), that do not unfold as homogeneously or “sequentially” as generally thought. Alternatively, it might signal an adaptive change of cognitive strategy according to the task, or stimulus/prompt at hand (e.g., depending on salience of fixating response, or past experiences with the task). In short, people may adapt flexibly, with strategy use (e.g., memory retrieval vs. effortful associative search) varying across responses according to task context, stimulus salience, and experience, challenging the notion of a uniform ideation strategy at the individual level. An important direction for examining this hypothesis is therefore to identify typical response sequences (e.g., via mixture or latent-class modeling) that may reflect qualitatively distinct ideation strategies unfolding over ideation sequences.

Together, this pattern of findings resembles the “reliability paradox” (Hedge et al., 2018; see also Cronbach, 1957; Karvelis & Diaconescu, 2025), whereby effects that are robust and reproducible at the aggregate level nevertheless show poor reliability as measures of inter-individual differences. From this perspective, the SOE appears “robust” for capturing general (i.e., group-level) ideational dynamics, yet limited for measuring individuals on an enduring creative problem-solving tendency. In sum, the robustness of an aggregate effect does not imply reliability in measuring individual SOEs. As emphasized by Hedge et al. (2018), experimental paradigms tend to become established precisely because they minimize between-person variance while maximizing within-person regularities, yielding reproducible mean-level effects but little stable variance with which to rank individuals. Although the SOE can be consistently demonstrated at the group level, it captures little dependable between-person variance once stimulus and method-specific influences are considered (here reflected in the very low scoring and prompt stability of individual SOE slopes). From a psychometric perspective, the SOE may therefore be better understood as a within-task ideational dynamic elicited by DT tasks, rather than as a marker of a stable, “universal” trait-like ideation strategy. This carries important theoretical and practical implications: while the SOE is informative for characterizing general processes that unfold during idea generation, it does not presently support inferences about enduring individual differences, highlighting the need for future work examining how individual-level ideation dynamics can be measured with adequate reliability (cf. Kievit et al., 2013). 

### 4.3. Limitations and Future Directions

Although this study offers important insights into the SOE literature, its conclusions should be interpreted considering several limitations that help contextualize the findings and guide future research. A primary consideration is the reliance on a secondary dataset, limiting causal and methodological control. While the dataset was suitable for the analyses conducted here (multiple scoring approaches, multiple DT prompts and typical time on task; see Saretzki et al., 2024), some key limitations inherent to the original data collection constrain the generalizability of our findings. 

First, the sample available for this study was relatively small and homogeneous, and relied on only three prompts from a single DT task (i.e., AUT). Although the AUT is the most central (Barbot et al., 2026) and frequently used (Saretzki et al., 2024) operationalization of DT in creativity studies, many other DT tasks exist, including other modalities such as graphic, scientific, or social problem-solving. Can we expect a SOE in a graphic DT task and, if so, can it be reasonably interpreted in terms of well-established mechanisms such as the spreading activation of increasingly semantically distant concepts (Mednick, 1962)? Similarly, only three scoring methods were available in the dataset, whereas other scoring approaches remain unexplored (e.g., other subjective ratings of creativity criteria such as cleverness or surprise). 

Second, methodological constraints of the dataset warrant caution: because the original study was not explicitly designed to examine the SOE, it is unclear whether, for example, raters involved in the subjective judgment of creativity were blind to the serial order of responses. Indeed, this factor may influence the scoring of individual responses (see, e.g., Kenett et al., 2023; Raz et al., 2025), implicitly crediting more later responses, and therefore, increasing the likelihood of observing an SOE. 

Third, the fixed 2.5 min time limit likely constrained ideational sequences, reducing the reliability of individual SOE slopes. Although ideation sequences were relatively short, the use of empirical Bayes shrinkage estimators helps mitigate the unreliability typically associated with limited observations. Thus, while longer sequences would be preferable, the dataset still provides a reasonable basis for examining inter-individual stability. Yet, studies with longer time-on-task would likely yield more stable estimates. 

Finally, only a few potential moderators were considered in the present study. Other methodological moderators of the SOE could be considered. In particular, beyond the solution space (Forthmann et al., 2020), the presence of salient, prominent, and fixating responses associated with a given task or stimulus could carry significant weight on the observation of the SOE. Additionally, the operationalization of “order” varies widely across SOE studies: from the full-sequence approaches used here (e.g., Gonthier & Besançon, 2024; Miroshnik & Shcherbakova, 2019), to segmentation by halves (Kaya & Acar, 2019; Runco, 1986), quarters (Wang et al., 2017), or time intervals (Beaty & Silvia, 2012), these choices may substantially affect observed effect sizes and open an additional research direction to examine. 

In sum, future studies purposely designed to address our research questions should be conducted to examine the generalization of our findings (regarding effect sizes and their moderators, inter-individual stability) to different tasks, scoring methods, and samples. Such studies should consider longer time-on-task to increase the length of ideation sequences, implement rigorous experimental controls (e.g., blinded scoring), and the inclusion of diverse task modalities, as well as a broader range of individual difference moderators, to more precisely isolate the mechanisms driving the SOE. 

Despite limitations, the present findings suggest several promising directions for future research. (1) The generally low SOE magnitudes observed here and in prior work underscore the need for systematic meta-analytic evaluation of effect sizes, which, however, may be challenged by the heterogeneous practices in estimating SOE and reporting effect sizes. (2) Variability in SOE strength across scoring methods, tasks, and stimuli indicates that not all creativity criteria exhibit a consistent SOE, highlighting its susceptibility to methodological influences that must be further examined. (3) Most critically, the lack of stability in inter-individual SOE differences across tasks and scoring methods suggests that an individual’s “creativity slope” is unlikely to reflect a reliable trait. This points to a potential “reliability paradox” (Hedge et al., 2018) that warrants closer examination of intra-individual DT ideation patterns to clarify whether this lack of inter-individual stability reflects psychometric limitations, rather than evidence that the SOE limitedly applies at the individual level. 

## Figures and Tables

**Table 1:** Parameter Estimates (and Corresponding Standard Errors) of the Linear Regression Model 1 across the Three Scoring Methods.

Effects	Model 1 (Subjective Judgment)			Model 1 (Frequency-Based)			Model 1 (Semantic Similarity)		
	Estimate (SE)	*F*	*p*	Estimate (SE)	*F*	*p*	Estimate (SE)	*F*	*p*
Fixed effects									
Intercept		3825.30	<.001		530.15	<.001		1780.49	<.001
Response order	0.15 (0.01)	109.76	<.001	−0.02 (0.002)	163.41	<.001	−0.01 (0.003)	4.21	.04
Task		33.94	<.001		22.84	<.001		82.13	<.001
Fluency		14.62	<.001		1.84	.18		18.42	<.001
Gender		11.40	<.001		4.77	.03		1.10	.30
Age		3.24	.07		0.31	.58		2.97	.09
AIC	11,040.07			−8434.99			−3248.86		
BIC	11,111.21			−8364.05			−3178.18		
*N_Observations_*	4763			4677			4567		
*N_Participants_*	202			202			202		
Variances									
Intercept (level 2)	0.07			0.0001			0.01		
Slope of resp. order (level 2)	0.01			0.0001			0.0003		
Residual (level 1)	0.55			0.01			0.03		

Note. To improve readability, only the regression coefficient and its standard error for the focal predictor (response order) are presented.

## Data Availability

The original raw data were collected as part of previous studies by Forthmann et al. (2017, 2020) and Forthmann and Doebler (2022), which are openly available in the Open Science Framework (OSF) repository at [https://osf.io/q5ap8 accessed on 2 February 2025].

## Notes

1 https://www.mdpi.com/journal/jintelligence/special_issues/J908U24N0Q, accessed on 1 January 2026.

2 To ensure a uniform directional interpretation across all three scoring methods, the slopes obtained from the subjective rating method were reverse-coded before consistency was estimated.

## Supplementary Materials

The following supporting information can be downloaded at: https://www.mdpi.com/article/10.3390/jintelligence14060100/s1. Table S1: Parameter Estimates (and Corresponding Standard Errors) of the Linear Regression Models across the Three Scoring Methods. Table S2: Parameter Estimates (and Corresponding Standard Errors) of the Quadratic Regression Models across the Three Scoring Methods. Figure S1: Distribution of Empirical Bayes Estimates across the Three Scoring Methods. Figure S2: Predicted Response Creativity by Response Order and Moderator Variable Interactions for Linear Regression Models across the Three Scoring Methods. Figure S3: Predicted Response Creativity by Response Order and Moderator Variable Interactions for Quadratic Regression Models across the Three Scoring Methods. Figure S4: Density plots for the three object-prompts.

## Abbreviations

The following abbreviations are used in this manuscript:

AUT	Alternate Use Task
DT	Divergent Thinking
ESM	Electronic Supplementary Materials
SOE	Serial Order Effect

## References

[B1-jintelligence-14-00100] Acar S., Abdulla Alabbasi A. M., Runco M. A., Beketayev K. (2019). Latency as a predictor of originality in divergent thinking. Thinking Skills and Creativity.

[B2-jintelligence-14-00100] Acar S., Runco M. A., Ogurlu U. (2018). The moderating influence of idea sequence: A re-analysis of the relationship between category switch and latency. Personality and Individual Differences.

[B3-jintelligence-14-00100] Ackerman P. L. (1987). Individual differences in skill learning: An integration of psychometric and information processing perspectives. Psychological Bulletin.

[B4-jintelligence-14-00100] Ahn P. H., Van Swol L. M., Lu R. M., Kim S. J., Park H., Moulder R. G. (2023). Innovative ideas desire earlier communication: Exploring reverse serial-order effect and liberating cognitive constraint for organizational problem-solving. Journal of Behavioral Decision Making.

[B5-jintelligence-14-00100] Bai H., Leseman P. P. M., Moerbeek M., Kroesbergen E. H., Mulder H. (2021). Serial order effect in divergent thinking in five- to six-year-olds: Individual differences as related to executive functions. Journal of Intelligence.

[B6-jintelligence-14-00100] Bai H., Mulder H., Moerbeek M., Leseman P. P. M., Kroesbergen E. H. (2024). The development of divergent thinking in 4- to 6-year-old children. Creativity Research Journal.

[B7-jintelligence-14-00100] Barbot B. (2018). The dynamics of creative ideation: Introducing a new assessment paradigm. Frontiers in Psychology.

[B8-jintelligence-14-00100] Barbot B., Cerda K., Teo T. (2020). Negative ideation in creative problem-solving is task-specific too: Evidences from a sample of incarcerated juveniles. Thinking Skills and Creativity.

[B9-jintelligence-14-00100] Barbot B., Krivulskaya S., Hein S., Reich J., Thuma P. E., Grigorenko E. L. (2016). Identifying learning patterns of children at risk for specific reading disability. Developmental Science.

[B10-jintelligence-14-00100] Barbot B., Naczenski L. M., Baas M., Stevenson C. E. (2026). Varieties of divergent thinking: A network analysis of Guilford, Merriefield and Cox (1961). Intelligence.

[B11-jintelligence-14-00100] Barbot B., Tinio P. P. (2015). Where is the “g” in “creativity”? A specialization-differentiation hypothesis. Frontiers in Human Neuroscience.

[B12-jintelligence-14-00100] Beaty R. E., Johnson D. R. (2021). Automating creativity assessment with SemDis: An open platform for computing semantic distance. Behavior Research Methods.

[B13-jintelligence-14-00100] Beaty R. E., Silvia P. J. (2012). Why do ideas get more creative across time? An executive interpretation of the serial order effect in divergent thinking tasks. Psychology of Aesthetics, Creativity, and the Arts.

[B14-jintelligence-14-00100] Ben-Shachar M. S., Lüdecke D., Makowski D. (2020). effectsize: Estimation of effect size indices and standardized parameters. Journal of Open Source Software.

[B15-jintelligence-14-00100] Butler A. B., Scherer L. L., Reiter-Palmon R. (2003). Effects of solution elicitation aids and need for cognition on the generation of solutions to ill-structured problems. Creativity Research Journal.

[B16-jintelligence-14-00100] Cassotti M., Agogué M., Camarda A., Houdé O., Borst G. (2016). Inhibitory control as a core process of creative problem solving and idea generation from childhood to adulthood. New Directions for Child and Adolescent Development.

[B17-jintelligence-14-00100] Cheng L., Hu W., Jia X., Runco M. A. (2016). The different role of cognitive inhibition in early versus late creative problem finding. Psychology of Aesthetics, Creativity, and the Arts.

[B18-jintelligence-14-00100] Christensen P. R., Guilford J. P., Wilson R. C. (1957). Relations of creative responses to working time and instructions. Journal of Experimental Psychology.

[B19-jintelligence-14-00100] Chrysikou E. G., Motyka K., Nigro C., Yang S.-I., Thompson-Schill S. L. (2016). Functional fixedness in creative thinking tasks depends on stimulus modality. Psychology of Aesthetics, Creativity, and the Arts.

[B20-jintelligence-14-00100] Cohen J. (1988). Statistical power analysis for the behavioral sciences.

[B21-jintelligence-14-00100] Cronbach L. J. (1957). The two disciplines of scientific psychology. American Psychologist.

[B22-jintelligence-14-00100] Dahlman S., Bäckström P., Bohlin G., Frans Ö. (2013). Cognitive abilities of street children: Low-SES Bolivian boys with and without experience of living in the street. Child Neuropsychology.

[B23-jintelligence-14-00100] de Chantal P.-L., Zielińska A., Lebuda I., Karwowski M. (2025). How do examples impact divergent thinking? The interplay between associative and executive processes. Psychology of Aesthetics, Creativity, and the Arts.

[B24-jintelligence-14-00100] Diamond A. (2013). Executive functions. Annual Review of Psychology.

[B25-jintelligence-14-00100] Forthmann B., Doebler P. (2022). Fifty years later and still working: Rediscovering Paulus et al.’s (1970) automated scoring of divergent thinking tests. Psychology of Aesthetics, Creativity, and the Arts.

[B26-jintelligence-14-00100] Forthmann B., Gerwig A., Holling H., Çelik P., Storme M., Lubart T. (2016). The be-creative effect in divergent thinking: The interplay of instruction and object frequency. Intelligence.

[B27-jintelligence-14-00100] Forthmann B., Holling H., Çelik P., Storme M., Lubart T. (2017). Typing speed as a confounding variable and the measurement of quality in divergent thinking. Creativity Research Journal.

[B28-jintelligence-14-00100] Forthmann B., Paek S. H., Dumas D., Barbot B., Holling H. (2020). Scrutinizing the basis of originality in divergent thinking tests: On the measurement precision of response propensity estimates. British Journal of Educational Psychology.

[B29-jintelligence-14-00100] Gilhooly K. J., Fioratou E., Anthony S. H., Wynn V. (2007). Divergent thinking: Strategies and executive involvement in generating novel uses for familiar objects. British Journal of Psychology.

[B30-jintelligence-14-00100] Golab D., Barbot B. (2024). Building narratives through empathy: The role of empathy mechanisms and associative thinking in creative writing. The Journal of Creative Behavior.

[B31-jintelligence-14-00100] Gonthier C., Besançon M. (2024). It is not always better to have more ideas: Serial order and the trade-off between fluency and elaboration in divergent thinking tasks. Psychology of Aesthetics, Creativity, and the Arts.

[B32-jintelligence-14-00100] Grissom N. M., Reyes T. M. (2019). Let’s call the whole thing off: Evaluating gender and sex differences in executive function. Neuropsychopharmacology.

[B33-jintelligence-14-00100] Guilford J. P. (1967). The nature of human intelligence.

[B34-jintelligence-14-00100] Harrington David M. (1975). Effects of explicit instructions to “be creative” on the psychological meaning of divergent thinking test scores1. Journal of Personality.

[B35-jintelligence-14-00100] Hass R. W. (2017). Tracking the dynamics of divergent thinking via semantic distance: Analytic methods and theoretical implications. Memory & Cognition.

[B36-jintelligence-14-00100] Hass R. W., Beaty R. E. (2018). Use or consequences: Probing the cognitive difference between two measures of divergent thinking. Frontiers in Psychology.

[B37-jintelligence-14-00100] Hedge C., Powell G., Sumner P. (2018). The reliability paradox: Why robust cognitive tasks do not produce reliable individual differences. Behavior Research Methods.

[B38-jintelligence-14-00100] Heinonen J., Numminen J., Hlushchuk Y., Antell H., Taatila V., Suomala J. (2016). Default mode and executive networks areas: Association with the serial order in divergent thinking. PLoS ONE.

[B39-jintelligence-14-00100] Hocevar D. (1979). A comparison of statistical infrequency and subjective judgment as criteria in the measurement of originality. Journal of Personality Assessment.

[B40-jintelligence-14-00100] Hubert K. F., Finch A., Zabelina D. (2025). Diminishing creative returns: Predicting optimal creative performance via individual differences in executive functioning. Psychology of Aesthetics, Creativity, and the Arts.

[B41-jintelligence-14-00100] Jendryczko D. (2024). Decomposing the true score variance in rated responses to divergent thinking-tasks for assessing creativity: A multitrait–multimethod analysis. Journal of Intelligence.

[B42-jintelligence-14-00100] Karvelis P., Diaconescu A. O. (2025). Clarifying the reliability paradox: Poor measurement reliability attenuates group differences. Frontiers in Psychology.

[B43-jintelligence-14-00100] Kaya F., Acar S. (2019). The impact of originality instructions on cognitive strategy use in divergent thinking. Thinking Skills and Creativity.

[B44-jintelligence-14-00100] Kenett Y. N., Gooz N., Ackerman R. (2023). The role of semantic associations as a metacognitive cue in creative idea generation. Journal of Intelligence.

[B45-jintelligence-14-00100] Kievit R., Frankenhuis W. E., Waldorp L., Borsboom D. (2013). Simpson’s paradox in psychological science: A practical guide. Frontiers in Psychology.

[B46-jintelligence-14-00100] Kleinkorres R., Forthmann B., Holling H. (2021). An experimental approach to investigate the involvement of cognitive load in divergent thinking. Journal of Intelligence.

[B47-jintelligence-14-00100] Koo T. K., Li M. Y. (2016). A guideline of selecting and reporting intraclass correlation coefficients for reliability research. Journal of Chiropractic Medicine.

[B48-jintelligence-14-00100] Kraus B., Cadle C., Simon-Dack S. (2019). EEG alpha activity is moderated by the serial order effect during divergent thinking. Biological Psychology.

[B49-jintelligence-14-00100] Lenth R. (2023). emmeans: Estimated marginal means, aka least-squares means *(Version 1.8.5)*.

[B50-jintelligence-14-00100] Lüdecke D. (2018). ggeffects: Tidy data frames of marginal effects from regression models. Journal of Open Source Software.

[B51-jintelligence-14-00100] Maldonado T., Orr J. M., Goen J. R. M., Bernard J. A. (2020). Age differences in the subcomponents of executive functioning. The Journals of Gerontology: Series B.

[B52-jintelligence-14-00100] Mednick S. (1962). The associative basis of the creative process. Psychological Review.

[B53-jintelligence-14-00100] Miroshnik K. G., Shcherbakova O. V. (2019). The proportion and creativity of “old” and “new” ideas: Are they related to fluid intelligence?. Intelligence.

[B54-jintelligence-14-00100] Myszkowski N., Storme M. (2025). Bayesian estimation of generalized log-linear poisson item response models for fluency scores using brms and stan. Journal of Intelligence.

[B55-jintelligence-14-00100] Nijstad B. A., De Dreu C. K., Rietzschel E. F., Baas M. (2010). The dual pathway to creativity model: Creative ideation as a function of flexibility and persistence. European Review of Social Psychology.

[B56-jintelligence-14-00100] Pellegrino G., Saretzki J., Benedek M. (2025). Controlling rater effects in divergent thinking assessment: An item response theory approach to individual response and snapshot scoring. Journal of Intelligence.

[B57-jintelligence-14-00100] Pinheiro J., Bates D., R Core Team (2025). nlme: Linear and nonlinear mixed effects models *(Version 3.1-169) [Computer software]*.

[B58-jintelligence-14-00100] Raudenbush S. W., Bryk A. S. (2002). Hierarchical linear models: Applications and data analysis methods.

[B59-jintelligence-14-00100] Raz T., Reiter-Palmon R., Kenett Y. N. (2025). The role of asking more complex questions in creative thinking. Psychology of Aesthetics, Creativity, and the Arts.

[B60-jintelligence-14-00100] Reiter-Palmon R., Forthmann B., Barbot B. (2019). Scoring divergent thinking tests: A review and systematic framework. Psychology of Aesthetics, Creativity, and the Arts.

[B61-jintelligence-14-00100] Revelle W. (2025). psych: Procedures for psychological, psychometric, and personality research *(Version 2.5.6) [Computer software]*.

[B62-jintelligence-14-00100] Runco M. A. (1986). Flexibility and originality in children’s divergent thinking. Journal of Psychology: Interdisciplinary and Applied.

[B63-jintelligence-14-00100] Runco M. A., Dow G., Smith W. R. (2006). Information, experience, and divergent thinking: An empirical test. Creativity Research Journal.

[B64-jintelligence-14-00100] Runco M. A., Illies J. J., Eisenman R. (2005). Creativity, originality, and appropriateness: What do explicit instructions tell us about their relationships?. The Journal of Creative Behavior.

[B65-jintelligence-14-00100] Said-Metwaly S., Fernández-Castilla B., Kyndt E., Van den Noortgate W. (2020). Testing conditions and creative performance: Meta-analyses of the impact of time limits and instructions. Psychology of Aesthetics, Creativity, and the Arts.

[B66-jintelligence-14-00100] Said-Metwaly S., Fernández-Castilla B., Kyndt E., Van den Noortgate W., Barbot B. (2021). Does the fourth-grade slump in creativity actually exist? A meta-analysis of the development of divergent thinking in school-age children and adolescents. Educational Psychology Review.

[B67-jintelligence-14-00100] Said-Metwaly S., Taylor C. L., Camarda A., Barbot B. (2022). Divergent thinking and creative achievement—How strong is the link? An updated meta-analysis. Psychology of Aesthetics, Creativity, and the Arts.

[B68-jintelligence-14-00100] Saretzki J., Forthmann B., Benedek M. (2024). A systematic quantitative review of divergent thinking assessments. Psychology of Aesthetics, Creativity, and the Arts.

[B69-jintelligence-14-00100] Satterthwaite F. E. (1946). An approximate distribution of estimates of variance components. Biometrics Bulletin.

[B70-jintelligence-14-00100] Sidi Y., Torgovitsky I., Soibelman D., Miron-Spektor E., Ackerman R. (2020). You may be more original than you think: Predictable biases in self-assessment of originality. Acta Psychologica.

[B71-jintelligence-14-00100] Silvia P. J., Winterstein B. P., Willse J. T., Barona C. M., Cram J. T., Hess K. I., Martinez J. L., Richard C. A. (2008). Assessing creativity with divergent thinking tasks: Exploring the reliability and validity of new subjective scoring methods. Psychology of Aesthetics, Creativity, and the Arts.

[B72-jintelligence-14-00100] Smith S. M., Ward T. B., Schumacher J. S. (1993). Constraining effects of examples in a creative generation task. Memory & Cognition.

[B73-jintelligence-14-00100] Taylor C. L., Barbot B. (2024). Dual pathways in creative writing processes. Psychology of Aesthetics, Creativity, and the Arts.

[B74-jintelligence-14-00100] Taylor C. L., Kaufman J. C., Barbot B. (2021). Measuring creative writing with the storyboard task: The role of effort and story length. The Journal of Creative Behavior.

[B75-jintelligence-14-00100] Taylor C. L., Said-Metwaly S., Camarda A., Barbot B. (2024). Gender differences and variability in creative ability: A systematic review and meta-analysis of the greater male variability hypothesis in creativity. Journal of Personality and Social Psychology.

[B76-jintelligence-14-00100] Torrance E. P. (1998). The torrance tests of creative thinking–norms—Technical manual research edition—Verbal tests, forms A and B—Figural Tests, forms A and B.

[B77-jintelligence-14-00100] Wallach M. A., Kogan N. (1965). Modes of thinking in young children.

[B78-jintelligence-14-00100] Wang M., Hao N., Ku Y., Grabner R. H., Fink A. (2017). Neural correlates of serial order effect in verbal divergent thinking. Neuropsychologia.

[B79-jintelligence-14-00100] Ward T. B. (1994). Structured imagination: The role of category structure in exemplar generation. Cognitive Psychology.

[B80-jintelligence-14-00100] Weiss S., Elmdust L. S., Goecke B. (2025). Small samples, big insights: A methodological comparison of estimation techniques for latent divergent thinking models. Journal of Intelligence.

[B81-jintelligence-14-00100] Wichmann F. A., Hill N. J. (2001). The psychometric function: I. Fitting, sampling, and goodness of fit. Perception & Psychophysics.

[B82-jintelligence-14-00100] Wilson R. C., Guilford J. P., Christensen P. R. (1953). The measurement of individual differences in originality. Psychological Bulletin.

[B83-jintelligence-14-00100] Zelazo P. D., Carlson S. M. (2012). Hot and cool executive function in childhood and adolescence: Development and plasticity. Child Development Perspectives.

