# Supplementary material for: “Robust” but Tiny: Methodological Influences and Inter-Individual (Un)Stability of the Serial Order Effect in Creativity
Source: J Intell. 2026 Jun 4;14(6):100. doi: 10.3390/jintelligence14060100 (PMC13300940; doi:10.3390/jintelligence14060100)

Electronic Supplementary Materials (ESM)

ESM1-Table S1

Parameter Estimates (and Corresponding Standard Errors) of the Linear Regression Models across the Three Scoring Methods

Scoring method	Effects	Model 1 Estimate (SE)	Model 1 *F*	Model 1 *p*	Model 2 Estimate (SE)	Model 2 *F*	Model 2 *p*	Model 3 Estimate (SE)	Model 3 *F*	Model 3 *p*	Model 4 Estimate (SE)	Model 4 *F*	Model 4 *p*	Model 5 Estimate (SE)	Model 5 *F*	Model 5 *p*	Model 6 Estimate (SE)	Model 6 *F*	Model 6 *p*
**Subjective judgment**	Fixed effects																		
	Intercept		3825.30	< .001		3835.86	< .001		3794.57	< .001		71.77	< .001		3572.72	< .001		3824.87	< .001
	Response order	**0.15 (0.01)**	**109.76**	**< .001**	**0.16 (0.02)**	**57.48**	**< .001**	**0.17 (0.02)**	**110.46**	**< .001**	**0.24 (0.04)**	**34.95**	**< .001**	**0.13 (0.03)**	**19.22**	**< .001**	**0.15 (0.01)**	**109.13**	**< .001**
	Task		33.94	< .001		33.61	< .001		33.91	< .001		32.57	< .001		33.87	<.001		33.94	< .001
	Fluency		14.62	< .001		14.52	< .001		17.55	< .001		0.01	.91		14.23	< .001		14.66	< .001
	Response order:Task		/	/		1.36	.26		/	/		/	/		/	/		/	/
	Garbage bag				0.16 (0.02)														
	Paper clip				0.18 (0.02)														
	Rope				0.13 (0.02)														
	Response order:Fluency		/	/		/	/	**-0.03 (0.01)**	**5.65**	**.02**		/	/		/	/		/	/
	Gender		11.40	< .001		11.49	< .001		11.85	< .001		11.74	< .001		8.90	.003		11..37	< .001
	Response order:Gender		/	/		/	/		/	/		/	/	0.03 (0.03)	0.92	.34		/	/
	Female													0.13 (0.03)					
	Male													0.16 (0.02)					
	Age		3.24	.07		3.27	.07		3.28	.07		3.33	.07		3.25	.07		3.39	.07
	Response order:Age		/	/		/	/		/	/		/	/		/	/	-0.01 (0.01)	0.17	.68
	Flexibility		/	/		/	/		/	/		0.47	.50		/	/		/	/
	Response order:Flexibility		/	/		/	/		/	/	**-0.01 (0.003)**	**5.12**	**.02**		/	/		/	/
	AIC		11040.07			11052.28			11043.53			11053.15			11046.13			11048.83	
	BIC		11111.21			11136.34			11121.13			11137.21			11123.73			11126.44	
	$N_{Observations}$		4763			4763			4763			4763			4763			4763	
	$N_{Participants}$		202			202			202			202			202			202	
	Variances																		
	Intercept (level 2)		0.07			0.07			0.07			0.07			0.07			0.07	
	Slope of response order (level 2)		0.01			0.01			0.01			0.01			0.01			0.01	
	Residual (level 1)		0.55			0.55			0.55			0.55			0.55			0.55	
**Frequency-based scoring**	Fixed effects																		
	Intercept		530.15	< .001		533.08	< .001		471.12	< .001		1.19	.28		529.35	< .001		530.86	< .001
	Response order	**-0.02 (0.002)**	**163.41**	**< .001**	**-0.02 (0.003)**	**81.86**	**< .001**	**-0.03 (0.002)**	**209.87**	**< .001**	**-0.05 (0.01)**	**109.34**	**< .001**	**-0.02 (0.003)**	**48.52**	**< .001**	**-0.02 (0.002)**	**163.86**	**< .001**
	Task		22.84	< .001		20.59	< .001		21.98	< .001		22.24	< .001		22.82	< .001		22.60	< .001
	Fluency		1.84	.18		1.85	.17		1.45	.22		0.68	.41		1.80	.18		1.83	.18
	Response order:Task		/	/		**12.09**	**< .001**		/	/		/	/		/	/		/	/
	Garbage bag				-0.024 (0.003)														
	Paper clip				-0.035 (0.003)														
	Rope				-0.017 (0.003)														
	Response order:Fluency		/	/		/	/	**0.01 (0.001)**	**38.44**	**< .001**		/	/		/	/		/	/
	Gender		4.77	.03		5.06	.03		5.18	.02		4.60	.03		4.74	.03		4.62	.03
	Response order:Gender		/	/		/	/		/	/		/	/	0.0003 (0.004)	0.01	.94		/	/
	Female													-0.02 (0.003)					
	Male													-0.02 (0.002)					
	Age		0.31	.58		0.69	.41		0.03	.86		0.22	.64		.30	.58		0.002	.97
	Response order:Age		/	/		/	/		/	/		/	/		/	/	0.002 (0.001)	1.10	.29
	Flexibility		/	/		/	/		/	/		0.96	.33		/	/		/	/
	Response order:Flexibility		/	/		/	/		/	/	**0.002 (0.001)**	**34.18**	**< .001**		/	/		/	/
	AIC		-8434.99			-8435.80			-8450.40			-8439.94			-8423.56			-8422.77	
	BIC		-8364.05			-8351.97			-8373.02			-8356.11			-8346.17			-8345.39	
	$N_{Observations}$		4677			4677			4677			4677			4677			4677	
	$N_{Participants}$		202			202			202			202			202			202	
	Variances																		
	Intercept (level 2)		0.0001			0.0001			0.0001			0.0001			0.0001			0.0001	
	Slope of response order (level 2)		0.0001			0.0001			0.000004			0.00003			0.0001			0.0001	
	Residual (level 1)		0.01			0.01			0.01			0.01			0.01			0.01	
**Semantic similarity**	Fixed effects																		
	Intercept		1780.49	< .001		1783.67	< .001		1748.48	< .001		103.67	< .001		1764.42	< .001		1780.80	< .001
	Response order	**-0.01 (0.003)**	**4.21**	**.04**	-0.0001 (0.005)	0.0002	.99	-0.01 (0.004)	5.07	.02	-0.01 (0.01)	1.04	.31	0.001 (0.01)	0.02	.88	**-0.01 (0.003)**	**4.49**	**.03**
	Task		82.13	< .001		82.97	< .001		82.27	< .001		77.15	< .001		82.31	< .001		82.16	< .001
	Fluency		18.42	< .001		18.33	< .001		19.01	< .001		13.82	< .001		17.95	< .001		18.63	< .001
	Response order:Task		/	/		2.04	.13		/	/		/	/		/	/		/	/
	Garbage bag				-0.0001 (0.005)														
	Paper clip				-0.01 (0.005)														
	Rope				-0.01 (0.003)														
	Response order:Fluency		/	/		/	/	0.0024 (0.003)	0.86	.35		/	/		/	/		/	/
	Gender		1.10	.30		5.06	.03		1.06	.30		1.01	.32		1.54	.22		1.13	.29
	Response order:Gender		/	/		/	/		/	/		/	/	-0.01(0.01)	1.82	.18		/	/
	Female													0.001 (0.007)					
	Male													-0.01 (0.004)					
	Age		2.97	.09		0.69	.41		2.99	.09		2.68	.10		3.01	.08		3.19	.08
	Response order:Age		/	/		/	/		/	/		/	/		/	/	0.01 (0.003)	3.55	.06
	Flexibility		/	/		/	/		/	/		9.73	.002		/	/		/	/
	Response order:Flexibility		/	/		/	/		/	/	0.0002 (0.001)	0.10	.75		/	/		/	/
	AIC		-3248.86			-3231.99			-3237.64			-3234.59			-3240.70			-3240.38	
	BIC		-3178.18			-3148.47			-3160.55			-3151.07			-3163.60			-3163.29	
	$N_{Observations}$		4567			4567			4567			4567			4567			4567	
	$N_{Participants}$		202			202			202			202			202			202	
	**Variances**																		
	Intercept (level 2)		0.01			0.01			0.01			0.01			0.01			0.01	
	Slope of response order (level 2)		0.0003			0.0003			0.0002			0.0002			0.0003			0.0002	
	Residual (level 1)		0.03			0.03			0.03			0.03			0.03			0.03	

Note. Model 1 = model with the main effect (response order); Model 2 = interaction with task; Model 3 = interaction with fluency; Model 4 = interaction with flexibility; Model 5 = interaction with age; Model 6 = interaction with age. For categorical moderator variables, regression coefficient estimates are reported for each category. A slash (/) indicates that the predictor was not included in the model. Significant main or moderating effects are shown in bold.

Electronic Supplementary Materials (ESM)

ESM2-Table S2

Parameter Estimates (and Corresponding Standard Errors) of the Quadratic Regression Models across the Three Scoring Methods

Scoring method	Effects		Model 8			Model 9			Model 10			Model 11			Model 12	
		Estimate (SE)	*F*	*p*	Estimate (SE)	*F*	*p*	Estimate (SE)	*F*	*p*	Estimate (SE)	*F*	*p*	Estimate (SE)	*F*	*p*
	Fixed effects															
	Intercept		3607.43	< .001		3766.25	< .001		3740.74	< .001		3227.91	< .001		3790.62	< .001
	Response order	**0.18 (0.02)**	**53.01**	**< .001**	**0.17 (0.02)**	**111.35**	**< .001**	**0.17 (0.02)**	**109.22**	**< .001**	**0.15 (0.03)**	**24.07**	**< .001**	**0.18 (0.02)**	**128.68**	**< .001**
	Response order$^{\wedge 2}$	-0.02 (0.01)	3.47	.06	**-0.07 (0.02)**	**21.63**	**< .001**	**-0.08 (0.02)**	**23.78**	**< .001**	**-0.05 (0.02)**	**5.72**	**.02**	**-0.04 (0.01)**	**18.85**	**< .001**
	Task		17.82	< .001		33.30	< .001		32.64	< .001		33.81	<.001		34.08	< .001
	Fluency		13.30	< .001		13.56	< .001		0.04	.84		12.66	< .001		13.23	< .001
	Response order:Task		0.54	.58		/	/		/	/		/	/		/	/
	Response order$^{\wedge 2}$:Task		1.16	.31		/	/		/	/		/	/		/	/
	Garbage bag	-0.02 (0.02)														
	Paper clip	-0.06 (0.02)														
	Rope	-0.05 (0.01)														
	Response order:Fluency		/	/	0.01 (0.02)	0.53	.47		/	/		/	/		/	/
	Response order$^{\wedge 2}$:Fluency		/	/	**0.02 (0.01)**	**6.19**	**.01**		/	/		/	/		/	/
	Gender		11.86	< .001		11.63	< .001		11.46	< .001		8.86	.003		11.81	< .001
	Response order:Gender		/	/		/	/		/	/	0.04 (0.03)	1.25	.26		/	/
	Response order$^{\wedge 2}$:Gender		/	/		/	/		/	/	0.01 (0.02)	0.32	.57		/	/
	Female										-0.05 (0.02)					
**Subjective judgment**	Male										-0.04 (0.01)					
	Age		3.34	.07		3.29	.07		3.39	.07		3.25	.07		2.29	.13
	Response order:Age		/	/		/	/		/	/		/	/	0.01 (0.02)	0.11	.74
	Response order$^{\wedge 2}$:Age		/	/		/	/		/	/		/	/	-0.01 (0.01)	0.52	.47
	Flexibility		/	/		/	/		0.54	.46		/	/		/	/
	Response order:Flexibility		/	/		/	/	0.02 (0.02)	0.82	.37		/	/		/	/
	Response order$^{\wedge 2}$:Flexibility		/	/		/	/	**0.02 (0.01)**	**7.06**	**.01**		/	/		/	/
	AIC		11057.50			11040.98			11043.17			11042.79			11048.42	
	BIC		11160.96			11131.51			11140.17			11133.32			11138.95	
	$N_{Observations}$		4763			4763			4763			4763			4763	
	$N_{Participants}$		202			202			202			202			202	
	**Variances**															
	Intercept (level 2)		0.07			0.07			0.07			0.07			0.07	
	Slope of response order (level 2)		0.01			0.01			0.004			0.01			0.01	
	Residual (level 1)		0.55			0.55			0.55			0.55			0.55	
	Fixed effects															
	Intercept		373.57	< .001		368.06	< .001		367.85	< .001		299.34	< .001		404.32	< .001
	Response order	**-0.03 (0.003)**	**108.93**	**< .001**	**-0.03 (0.002)**	**225.24**	**< .001**	**-0.03 (0.002)**	**223.72**	**< .001**	**-0.03 (0.004)**	**60.86**	**< .001**	**-0.03 (0.002)**	**235.89**	**< .001**
	Response order$^{\wedge 2}$	**0.01 (0.002)**	**35.11**	**< .001**	**0.01 (0.002)**	**42.60**	**< .001**	**0.01 (0.002)**	**45.11**	**< .001**	**0.01 (0.003)**	**20.33**	**< .001**	**0.01 (0.001)**	**72.83**	**< .001**
	Task		7.23	< .001		22.38	< .001		22.72	< .001		22.12	< .001		22.35	< .001
	Fluency		0.26	.61		0.001	.98		1.63	.20		0.37	.54		0.43	.51
	Response order:Task		**9.84**	**< .001**		/	/		/	/		/	/		/	/
	Response order$^{\wedge 2}$:Task		2.25	.11		/	/		/	/		/	/		/	/
	Garbage bag	0.01 (0.002)														
	Paper clip	0.01 (0.002)														
	Rope	0.01 (0.002)														
	Response order:Fluency		/	/	-0.001 (0.002)	0.15	.70		/	/		/	/		/	/
	Response order$^{\wedge 2}$:Fluency		/	/	**-0.002 (0.001)**	**5.88**	**.02**		/	/		/	/		/	/
	Gender		5.53	.02		5.20	.02		5.12	.02		5.33	.02		4.50	.04
	Response order:Gender		/	/		/	/		/	/	-0.002 (0.004)	1.25	.26		/	/
	Response order$^{\wedge 2}$:Gender		/	/		/	/		/	/	-0.003 (0.003)	0.32	.57		/	/
**Frequency-based scoring**	Female										0.01 (0.003)					
	Male										0.01 (0.001)					
	Age		0.05	.83		0.01	.91		0.03	.86		0.02	.88		1.46	.23
	Response order:Age		/	/		/	/		/	/		/	/	0.004 (0.002)	3.83	.05
	Response order$^{\wedge 2}$:Age		/	/		/	/		/	/		/	/	**-0.002 (0.001)**	**4.94**	**.03**
	Flexibility		/	/		/	/		1.65	.20		/	/		/	/
	Response order:Flexibility		/	/		/	/	-0.001 (0.002)	0.13	.72		/	/		/	/
	Response order$^{\wedge 2}$:Flexibility		/	/		/	/	**-0.002 (0.001)**	**6.66**	**.01**		/	/		/	/
	AIC		-8459.64			-8467.36			-8461.24			-8466.25			-8468.46	
	BIC		-8356.48			-8377.08			-8364.52			-8375.98			-8378.18	
	$N_{Observations}$		4677			4677			4677			4677			4677	
	$N_{Participants}$		202			202			202			202			202	
	**Variances**															
	Intercept (level 2)		0.0001			0.0001			0.0001			0.0001			0.0001	
	Slope of response order (level 2)		0.000004			0.000002			0.000001			0.000001			0.00003	
	Residual (level 1)		0.01			0.01			0.01			0.01			0.01	
	Fixed effects															
	Intercept		1705.27	< .001		1706.92	< .001		1692.94	< .001		1596.85	< .001		1731.02	< .001
	Response order	0.000004 (0.01)	0.000001	.99	**-0.01 (0.004)**	**4.72**	**.03**	-0.01 (0.004)	3.42	.06	-001 (0.007)	0.03	.87	**-0.01 (0.003)**	**5.64**	**.02**
	Response order$^{\wedge 2}$	0.0004 (0.003)	0.03	.87	0.004 (0.004)	1.59	.21	0.01 (0.004)	2.59	.11	0.01 (0.005)	1.50	.22	0.002 (0.002)	1.23	.27
	Task		59.56	< .001		82.66	< .001		77.34	< .001		82.65	< .001		82.26	< .001
	Fluency		17.67	< .001		17.82	< .001		13.62	< .002		16.68	< .001		17.66	< .001
	Response order:Task		2.12	.12		/	/		/	/		/	/		/	/
Semantic similarity	Response order$^{\wedge 2}$:Task		.40	.67		/	/		/	/		/	/		/	/
	Garbage bag	0.0004 (0.003)														
	Paper clip	0.004 (0.004)														
	Rope	0.002 (0.003)														
	Response order:Fluency		/	/	0.001 (0.004)	0.08	.78		/	/		/	/		/	/
	Response order$^{\wedge 2}$:Fluency		/	/	-0.002 (0.001)	1.18	.28		/	/		/	/		/	/
	Gender		1.09	.30		1.08	.30		1.03	.31		0.99	.32		1.10	.30
	Response order:Gender		/	/		/	/		/	/	-0.01 (0.008)	1.40	.24		/	/
	Response order$^{\wedge 2}$:Gender		/	/		/	/		/	/	-0.004 (0.01)	0.55	.46		/	/

Electronic Supplementary Materials (ESM)

Female									0.01 (0.005)					
Male									0.002 (0.002)					
Age	3.04	.08		2.98	.09		2.67	.10		3.08	.08		3.08	.08
Response order:Age	/	/		/	/		/	/		/	/	0.01 (0.003)	2.22	.14
Response order^2:Age	/	/		/	/		/	/		/	/	-0.0002 (0.002)	0.01	.93
Flexibility	/	/		/	/		9.49	.002		/	/		/	/
Response order:Flexibility	/	/		/	/	-0.001 (0.004)	0.12	.73		/	/		/	/
Response order^2:Flexibility	/	/		/	/	-0.002 (0.002)	1.52	.22		/	/		/	/
AIC	-3199.18			-3214.30			-3217.48			-3219.79			-3216.23	
BIC	-3096.39			-3124.36			-3121.12			-3129.85			-3126.29	
$N_{Observations}$	4567			4567			4567			4567			4567	
$N_{Participants}$	202			202			202			202			202	
Variances														
Intercept (level 2)	0.01			0.01			0.01			0.01			0.01	
Slope of response order (level 2)	0.0002			0.0003			0.0003			0.0002			0.0002	
Residual (level 1)	0.03			0.03			0.03			0.03			0.03	

Note. Model 7 = model with the main effects (response order and response order^2); Model 8 = interaction with task; Model 9 = interaction with fluency; Model 10 = interaction with flexibility; Model 11 = interaction with gender; Model 12 = interaction with age. For categorical moderator variables, regression coefficient estimates are reported for each category. A slash (/) indicates that the predictor was not included in the model. Significant main or moderating effects are shown in bold.

Electronic Supplementary Materials (ESM)

ESM 3-Figure S1

Distribution of Empirical Bayes Estimates across the Three Scoring Methods

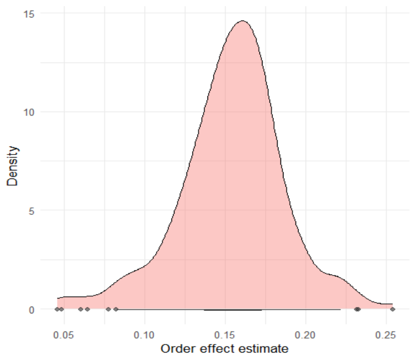

(B) Frequency-based scoring

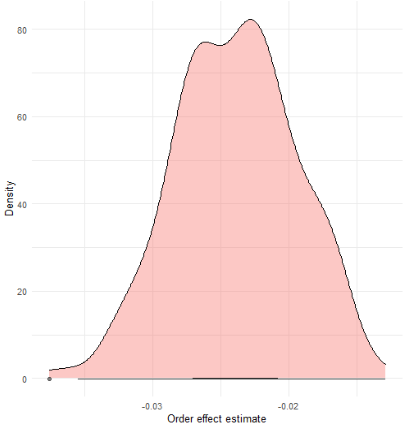

(C) Semantic similarity

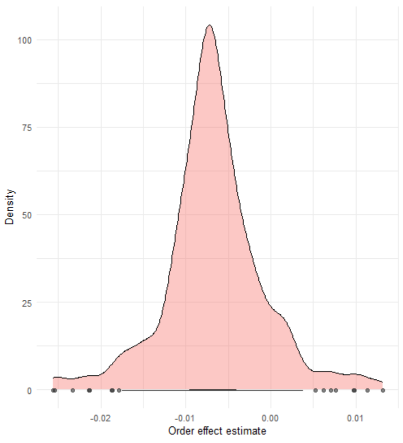

Electronic Supplementary Materials (ESM)

ESM4-Figure S2

Predicted Response Creativity by Response Order and Moderator Variable Interactions for Linear Regression Models across the Three Scoring Methods

(A) Subjective ratings

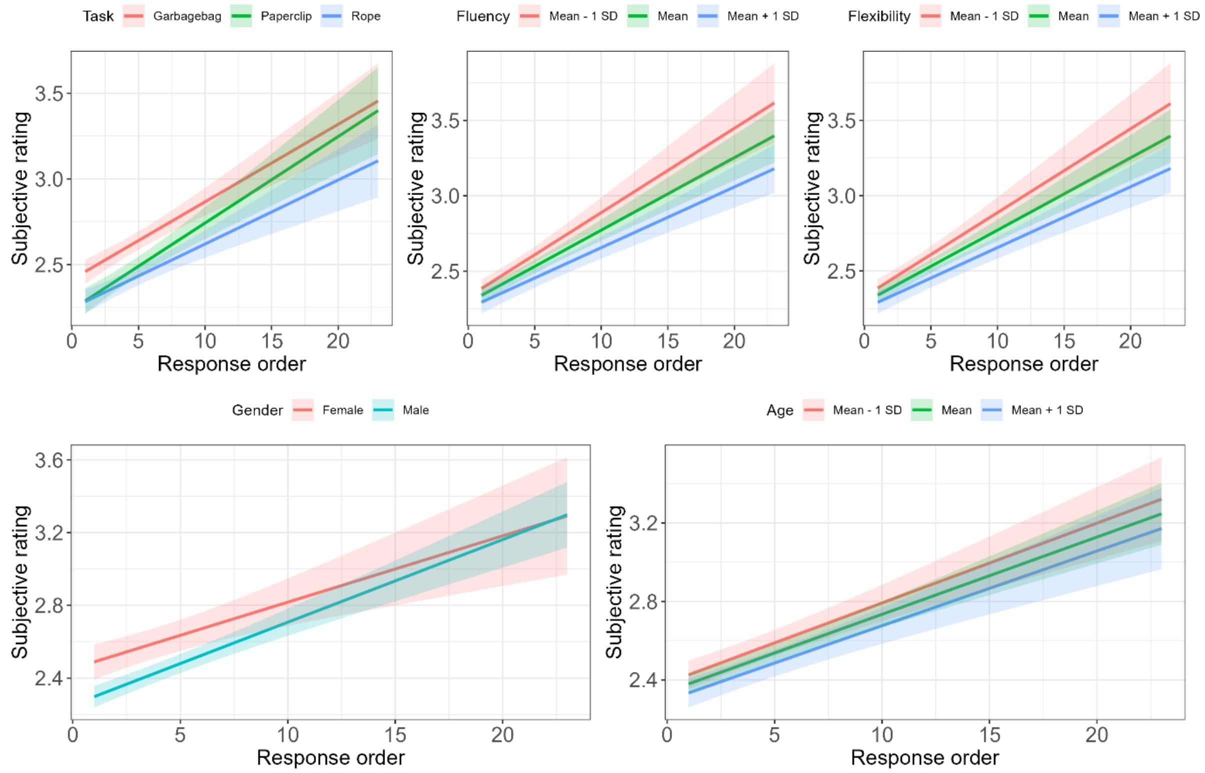

(B) Frequency-based scoring

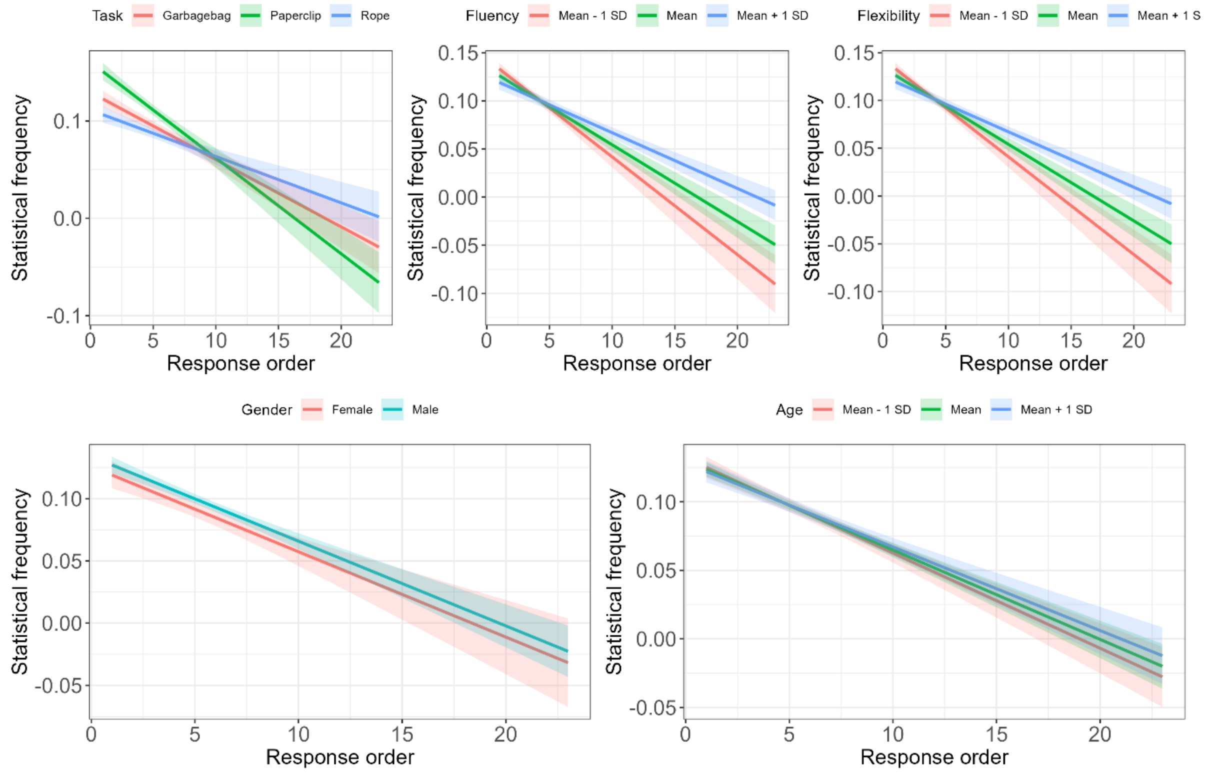

Electronic Supplementary Materials (ESM)

(C) Semantic similarity

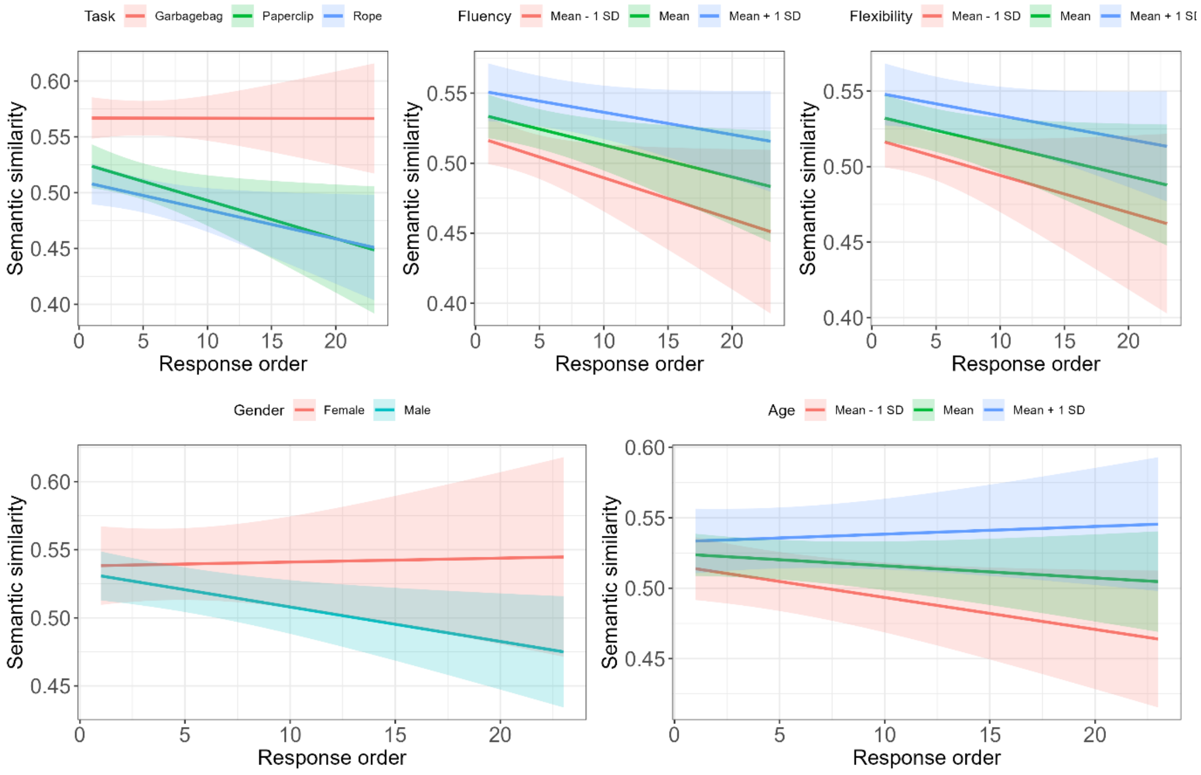

Electronic Supplementary Materials (ESM)

ESM5-Figure S3

Predicted Response Creativity by Response Order and Moderator Variable Interactions for Quadratic Regression Models across the Three Scoring Methods

(A) Subjective ratings

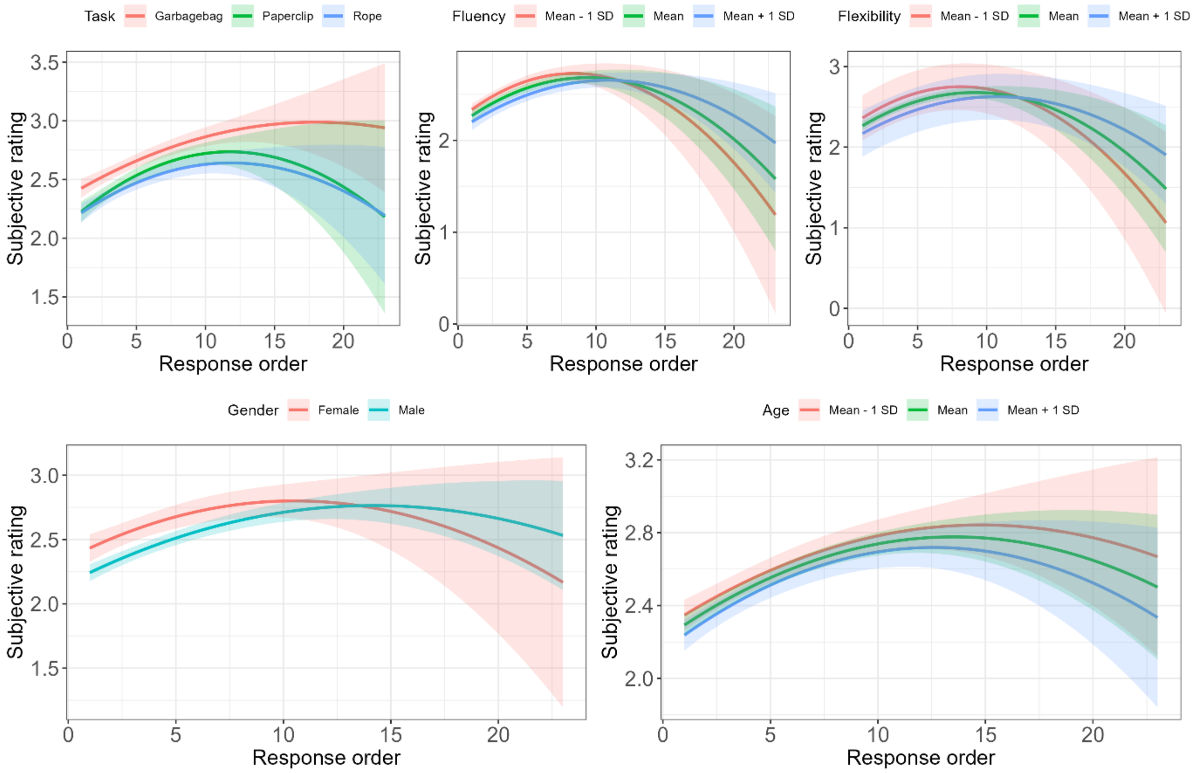

(B) Frequency-based scoring

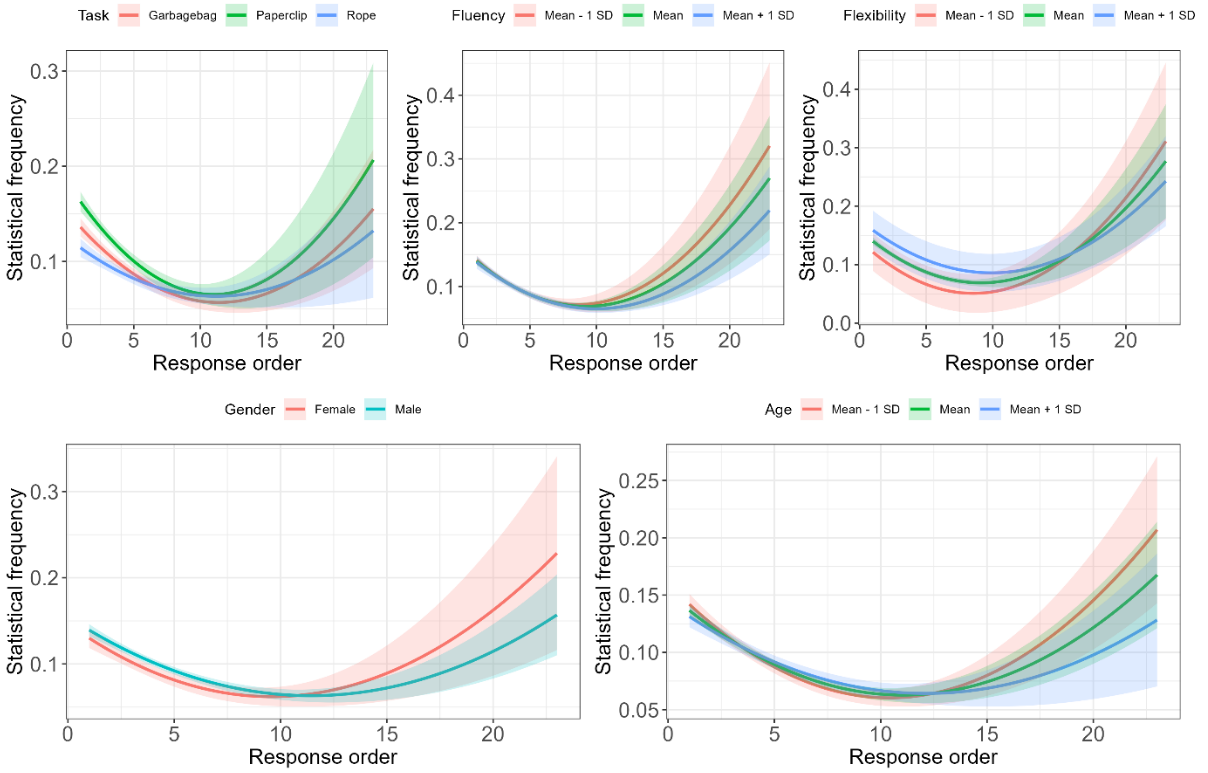

Electronic Supplementary Materials (ESM)

(C) Semantic similarity

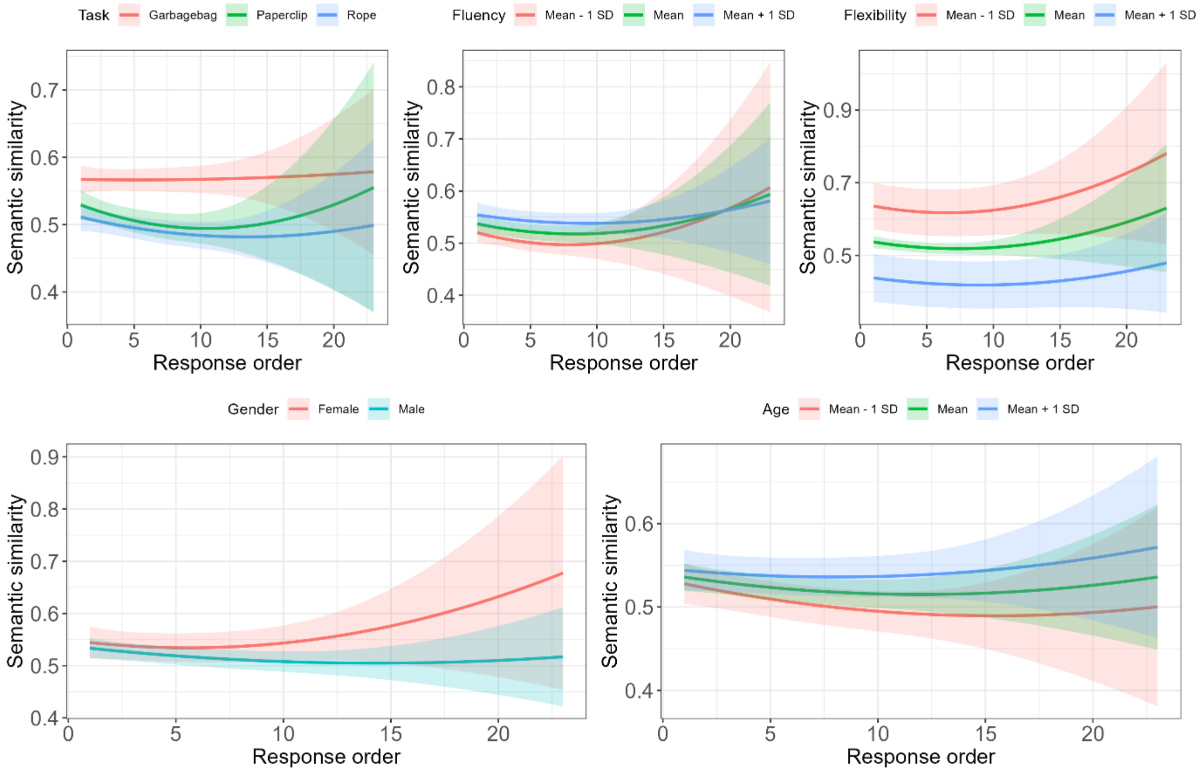

ESM6-Figure S4

Density plots for the three object-prompts

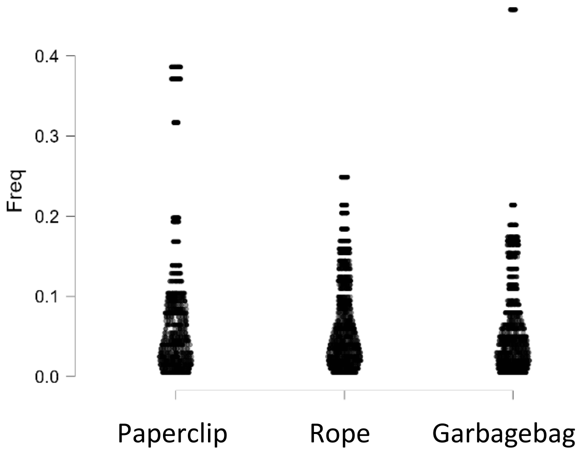

Supplement: Supplementary file 1 [file jintelligence-14-00100-s001.zip › jintelligence-4089624-supplementary.pdf]
